# Iron Accumulation and Lipid Peroxidation in the Aging Retina: Implication of Ferroptosis in Age-Related Macular Degeneration

**DOI:** 10.14336/AD.2020.0912

**Published:** 2021-04-01

**Authors:** Tantai Zhao, Xiaojian Guo, Yun Sun

**Affiliations:** ^1^Department of Ophthalmology, the Second Xiangya Hospital, Central South University, Changsha, Hunan, China.; ^2^Hunan Clinical Research Center of Ophthalmic Disease, Changsha, Hunan, China.

**Keywords:** iron, lipid peroxidation, ferroptosis, retina, age-related macular degeneration

## Abstract

Iron is an essential component in many biological processes in the human body. It is critical for the visual phototransduction cascade in the retina. However, excess iron can be toxic. Iron accumulation and reduced efficiency of intracellular antioxidative defense systems predispose the aging retina to oxidative stress-induced cell death. Age-related macular degeneration (AMD) is characterized by retinal iron accumulation and lipid peroxidation. The mechanisms underlying AMD include oxidative stress-mediated death of retinal pigment epithelium (RPE) cells and subsequent death of retinal photoreceptors. Understanding the mechanism of the disruption of iron and redox homeostasis in the aging retina and AMD is crucial to decipher these mechanisms of cell death and AMD pathogenesis. The mechanisms of retinal cell death in AMD are an area of active investigation; previous studies have proposed several types of cell death as major mechanisms. Ferroptosis, a newly discovered programmed cell death pathway, has been associated with the pathogenesis of several neurodegenerative diseases. Ferroptosis is initiated by lipid peroxidation and is characterized by iron-dependent accumulation. In this review, we provide an overview of the mechanisms of iron accumulation and lipid peroxidation in the aging retina and AMD, with an emphasis on ferroptosis.

Iron is the most abundant redox-active heavy metal and is indispensable for several biological processes [1]. Iron levels in the human body are maintained by precise uptake from the diet [2], with no mechanism for active iron excretion [3]. As a result, iron tends to accumulate in certain tissues during aging [4].

Iron accumulation in the aging brain has been documented in multiple neurodegenerative diseases [2]. Increased iron levels have been associated with more severe disease in patients with Alzheimer's and Parkinson's disease [5]. Iron accumulates in the aging retina and has been implicated in the pathogenesis of age-related macular degeneration (AMD) [4, 6].

AMD is the leading cause of irreversible blindness in developed countries among people aged 65 years and above [7]. The etiology of AMD is unknown, but age is the most consistent risk factor [8]. Elevated oxidative stress and lipid peroxidation also contribute to AMD progression [9]. Specifically, solar irradiation exposes the retina to oxidative stress-mediated damage. High concentrations of polyunsaturated fatty acids (PUFAs) in the photoreceptor outer segments are a major source of intracellular reactive oxygen species (ROS), which make the retina particularly vulnerable to oxidative stress-mediated damage [9]. Accordingly, the retina requires extraordinary antioxidant protection [10]. Indeed, the retina possesses several intracellular anti-oxidative mechanisms, including glutathione (GSH) and glutathione peroxidase (GPX), to minimize oxidative damage. The efficiency of these redox systems declines significantly with age, which further predisposes the retina to oxidative stress [11].

Both oxidative stress-mediated death of retinal pigment epithelium (RPE) cells and subsequent death of photoreceptors have been reported in AMD [12]. Previous studies considered apoptosis to be the major mechanism for RPE/photoreceptor cell death [13]. However, increasing evidence suggests that other types of regulated cell death (e.g., pyroptosis, necroptosis, and autophagy) also contribute to AMD [13-17].

Ferroptosis is a newly discovered, iron-dependent, regulated cell death pathway. It has been implicated in neurodegeneration, ischemia-reperfusion injury, and myocardial infarction [18]. It is initiated by lipid peroxidation and characterized by iron-dependent accumulation, clearly distinguished from apoptosis and other regulated cell death pathways in both morphology and mechanism [19-22]. Cells undergoing ferroptosis appear similar to those undergoing necrosis. Common morphological features include dysmorphic small mitochondria with decreased cristae, membrane condensation, and outer membrane rupture [21, 23]. The ‘‘ballooning’’ phenotype is generally considered as the final morphological feature of ferroptotic cells, but has not been shown in all cell types with other key features of ferroptosis [24]. Similar to apoptosis, ferroptosis is highly regulated and thus could be targeted for therapeutic purposes, but specific pathways, particularly inflammatory responses, are distinct. In apoptosis, the integrity of the plasma membrane is preserved along with hydrolytic digestion of intracellular contents. Apoptotic bodies are engulfed by macrophages, resulting in an anti-inflammatory process. In contrast, ferroptosis is primarily pro-inflammatory due to plasma membrane rupture and the release of intracellular contents [25-27]. In a general sense, ferroptosis is a mechanism that protects cellular integrity under normal conditions, but leads to cell death when cellular integrity is compromised, whereas apoptosis represents a suicide mechanism that eliminates certain types of cells from the whole organism at specific time points [28].

Studies on iron accumulation and elevated lipid peroxidation in the aging retina, and their intimate role in ferroptosis, have implicated ferroptosis in AMD pathogenesis [29-31]. In this review, we summarize the current evidence for disturbed iron and redox homeostasis in the aging retina. We also provide a brief history and recent progress in our understanding of the mechanism of ferroptosis.

## Iron homeostasis in the normal and aging retina

### Iron import, storage, and export in the retina

**Figure 1.**
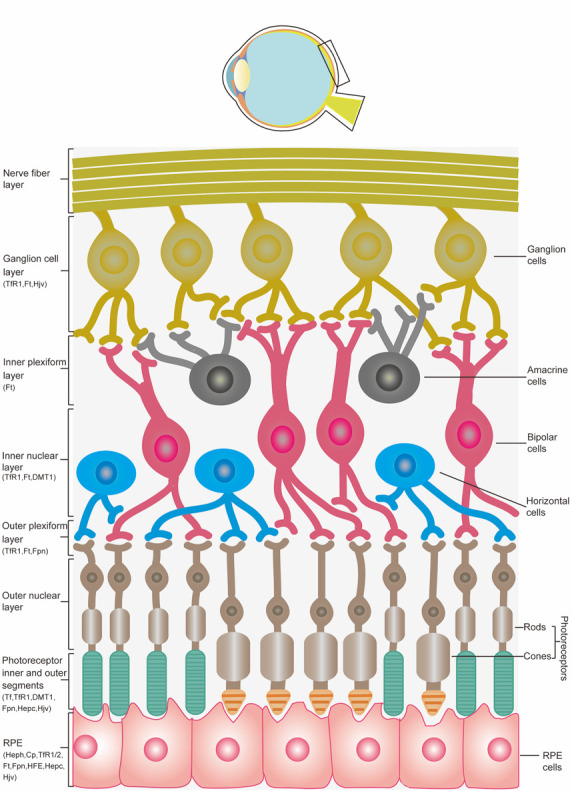
Illustration of the *bulbus oculi* with an enlarged view of the retinal layers and distribution of the proteins involved in iron metabolism in the retina.

The retina consists of the inner neural retina and the outer pigmented retina, or the RPE. The overall organization of the neural retina consists of a nerve fiber layer, ganglion cell layer, inner plexiform layer, inner nuclear layer, outer plexiform layer, outer nuclear layer, photoreceptor inner, and photoreceptor outer segments [32]. This organization is depicted in Figure 1.

More than 60% of total iron exists as heme iron. Unbound iron binds to the iron transport protein transferrin (Tf) in the form of ferric iron (Fe^3+^) *via* the Tf receptor (TfR) [33-35]. Ferroxidases, such as hephaestin (Heph) and ceruloplasmin (Cp) are produced in the retina and convert iron from the ferrous (Fe^2+^) to the ferric state [36]. The Tf-bound iron is absorbed into cells *via* internalization [37]. In the retina, Tf is predominantly found in the RPE and photoreceptors [38]. Cells regulate their intake of Tf-bound iron by modulating TfR1 expression on the cell surface [39]. TfR1, a type ǁ transmembrane glycoprotein, is involved in cellular iron uptake, whereas TfR2, a homolog of TfR1, also binds to and internalizes the Tf-Fe^3+^ complex but has lower affinity and more limited distribution than TfR1 [40]. TfR1 is expressed in the ganglion cell layer, inner nuclear layer, outer plexiform layer, photoreceptor inner segment, RPE, and choroid, whereas TfR2 is expressed in the RPE [41]. The specific location of TfR in the basolateral membrane of the RPE suggests a role in mediating the entry of Tf-bound iron from the choroidal circulation [42]. Iron import into the retina can occur *via* canonical Tf-TfR-mediated endocytosis [43, 44]. In iron-loaded retinas, Tf-bound iron import is downregulated. In contrast, Zip8 and Zip14 are upregulated and may take up increasing amounts of non-Tf-bound iron on vascular endothelial cells, exported as Fe2+ by ferroportin (Fpn), then imported directly into adjacent Müller cells by Zip8 or Zip14 without being oxidized or binding to Tf [45]. Once inside the Müller cells, iron can be redistributed within the retina [46]. In this case, changes in Zip8 and/or Zip14 may indicate loss of Müller cell and *vice versa* [47]. In addition, alpha-synuclein (α-syn), a ferrireductase in RPE cells, can facilitate the uptake of Tf-bound iron, but not non-Tf-bound iron [48].

Once imported into cells, Fe^3+^ dissociates from the Tf-TfR complex in the acidified endosome. The Fe^3+^-Tf-TfR complex is endocytosed in a clathrin-coated pit. Within the endosome, low pH (~5.6) causes the release of iron from the Fe^3+^-Tf-TfR complex [49-51]. Iron is then reduced to Fe^2+^ in the presence of ferrireductases, including the six-transmembrane epithelial antigen of prostate 3 (Steap3) [52] and duodenal cytochrome b (Dcytb) [50]. Fe^2+^is subsequently transported across the endosomal membrane into the cytoplasm by divalent metal transporter 1 (DMT1), a proton/Fe^2+^ symporter located on the endosomal membrane [45], that is present in rod bipolar cells, horizontal cells, and photoreceptor inner segments [42, 53]. Fe^2+^ may be stored in ferritin (Ft) [54], a cytoplasmic protein that is present as a dimer of heavy (FTH1, 21kDa), and light (FTL1, 19.5kDa) polypeptides [55]. The ferroxidase FTH1 oxidizes iron to facilitate its incorporation into Ft. FTL1 has no ferroxidase activity but promotes iron storage [55]. Unlike the Ft of insects, mammalian Ft lacks the signal peptide required for canonical endoplasmic reticulum-Golgi-mediated secretion [56]. Instead it is secreted through a non-canonical lysosomal secretion pathway [56, 57] by secretory lysosomes or exosomes [58]. Several cells within the retina express an FTL1 receptor called scavenger receptor member 5 (Scara5) [59], indicating that Ft secretion and uptake may occur in the retina. Scara5 has been reported to mediate the intracellular delivery of non-Tf-bound iron [60]. Other than storage, Ft functions as an antioxidant and a pro-angiogenesis factor. Increased expression of FTH1 in lens epithelial cells reportedly decreases intracellular iron levels and protects cells against oxidative stress [61]. Additionally, Ft plays a role in regulating angiogenesis [62]. Ft binds to an endogenous inhibitor of angiogenesis, a cleaved product of high molecular weight kininogen (HKa), with high affinity and antagonizes its antiangiogenic effects [62]. Ft expression in the retina is predominantly detectable in the inner segments of photoreceptors, RPE, choroid, outer plexiform layer, inner nuclear layer, and the ganglion cell layer [53]. In the inner nuclear layer, Ft is predominantly localized in axon terminals of rod bipolar cells, indicating that it may affect iron transport or storage in the synaptic terminal [42, 63].

**Figure 2.**
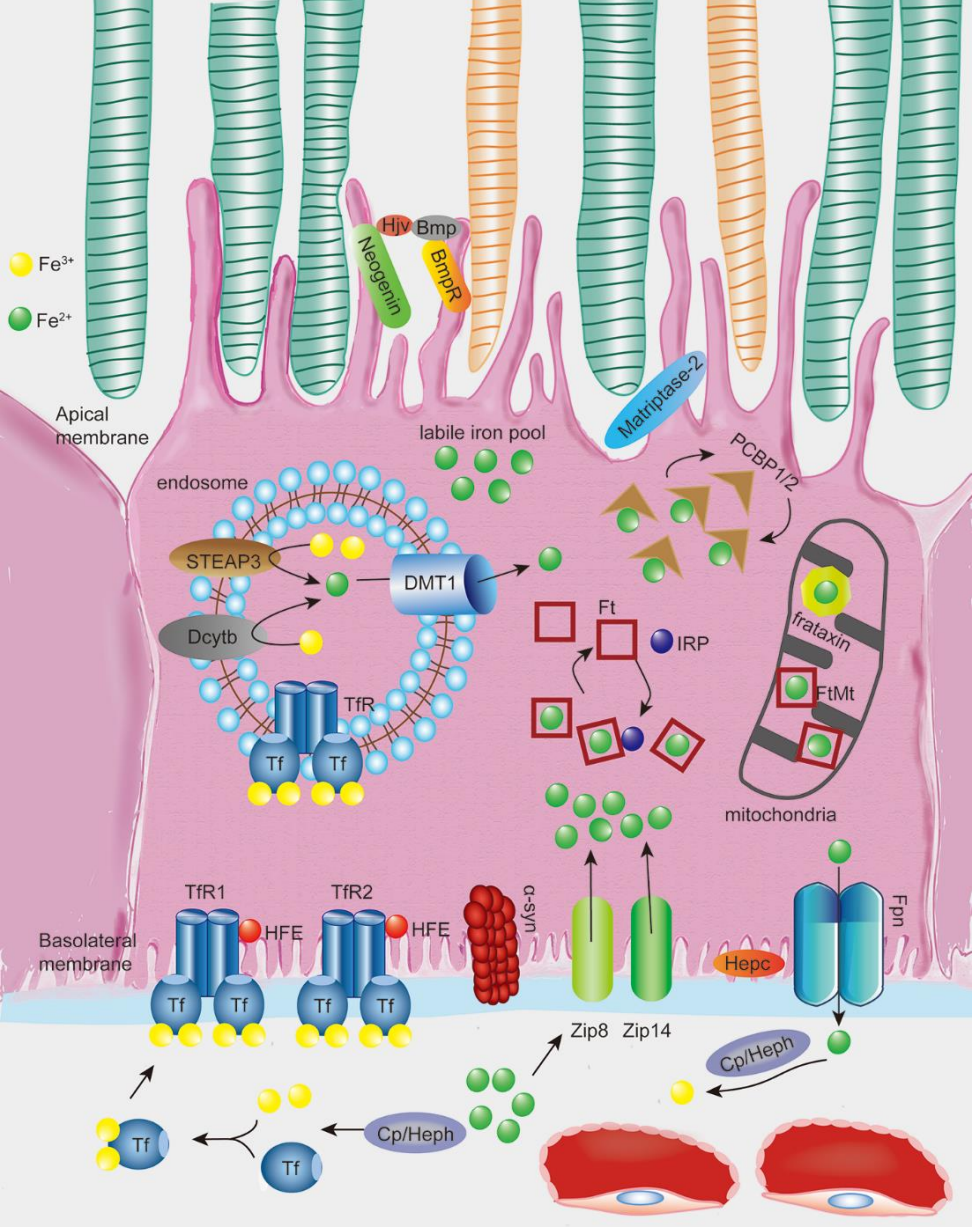
Illustration of the processes of iron uptake, storage, and efflux in the retinal pigment epithelium (RPE) cells. Two Fe^3+^atoms oxidized from Fe^2+^ by ferroxidase hephaestin (Heph) or ceruloplasmin (Cp) bind to the iron transport protein transferrin (Tf). Tf then binds to Tf receptor 1/2 (TfR1/2) in the basolateral membrane of RPE, modulated by HFE. In some conditions, retinal non-Tf bound iron import may be absorbed by Zip8 and/or Zip14 independent of the canonical Tf-TfR pathway without being oxidized. A ferrireductase, α-synuclein (α-syn) expressed in RPE cells can facilitate the uptake of Tf-bound iron but not non-Tf bound iron. Once inside cells, Fe^3+^ dissociates from the Tf-TfR complex in acidified endosomes followed by reduction of Fe^3+^ to Fe^2+^ catalyzed by ferrireductases, such as Steap3 and Dcytb, then transported across the endosomal membrane into the cytoplasm by the ferrous iron transporter DMT1. Fe^2+^ may then be stored into Ft in the cytoplasm or FtMt in the mitochondria, both of which are regulated by iron regulatory proteins (IRPs). Fe^2+^ is released from Ft through Ft degradation and is selectively recognized by the cytosolic and mitochondrial Fe^2+^ chaperones PCBPs1/2 and frataxin, respectively, then eventually utilized by diverse Fe^2+^-dependent proteins. The remaining Fe^2+^ enters the labile iron pool as the source of active-redox iron. Fe^2+^ that is not utilized or stored by the cell is exported by the transmembrane protein Fpn through post-translational regulation by hepcidin (Hepc). Fpn is also regulated by HFE, which is located at the basolateral membrane and by hemojuvelin (Hjv) and matriptase2, which are located at the apical membrane of the RPE through their regulatory effect towards Hepc. Fe^2+^ efflux is subsequently oxidized by ferroxidase Heph or Cp to facilitate the next cycle of iron uptake.

Iron released from degraded Ft in lysosomes is subsequently taken up by cells and enters the labile iron pool [64], which serves as a supply for storage, export, or metabolic utilization [65]. The cytosolic and mitochondrial iron chaperons, poly(rC)-binding proteins (PCBPs)1/2 and frataxin, respectively, selectively recognize Fe^2+^[66-68].

Unused or non-stored iron is exported through the exporter Fpn. This transmembrane protein is encoded by *SLC40A1* [69], recognizes Fe^2+^ as its substrate [70, 71], and is a known route for cellular iron efflux [72]. Ferroxidases Heph and Cp oxidize Fe^2+^ to its ferric state to facilitate iron export *via* Fpn and bind to extracellular Tf [73]. Fpn is present in the photoreceptor inner segments, outer plexiform layer, retinal vascular endothelial cells, Müller endfeet, and RPE [63]. Fpn localization in the RPE predominantly occurs in the basolateral membrane adjacent to the choroidal vasculature, suggesting that Fpn mediates iron export from the RPE into choroidal blood [53]. Fpn is predominantly localized on the abluminal surface of retinal vascular endothelial cells and participates in iron export across the blood-retinal barrier (BRB) [59]. Heph and Cp are co-expressed with Fpn in RPE and Müller cells to oxidize exported Fe^2+^, thereby facilitating the next cycle of iron uptake [74]. In summary, iron flux in the retina occurs through the retinal vasculature, exiting the retina through the basolateral RPE into the choroidal circulation or through the Müller cell endfeet and the internal limiting membrane into the vitreous [63] (Fig. 2).

### Iron-handling proteins in the retina

The intracellular iron concentration is regulated at three levels: controlling iron uptake by modulating TfR expression, modulating the labile iron pool (by regulating Ft expression), and regulating iron export (by modulating Fpn expression) [75-78] (Fig. 2).

### Regulation of TfR expression

HFE, a major histocompatibility complex (MHC) human leukocyte antigen (HLA) class I-like protein involved in iron homeostasis, recognizes the saturation of Tf-bound iron by interacting with TfR1 and TfR2 [41, 53]. HFE binds to TfR1 in addition to the Fe^3+^-Tf-TfR pathway on the cell surface; however, on sites that only partially overlap with the ones contacted by Tf, to reduce Fe^3+^-Tf binding affinity [79]. The Tf-contact area over the helical domain allows simultaneous binding, and thus the formation of a ternary HFE/TfR1/Fe^3+^-Tf complex [80]. HFE/Fe^3+^-Tf competition for cellular entry through TfR1 provides a dynamic tool to control intracellular iron concentration [79, 81]. Conversely, the TfR2/HFE complex is important for transcriptional regulation of the peptide hormone hepcidin (Hepc) [82] and inhibits iron uptake by triggering the degradation of the iron exporter Fpn [83]. HFE and TfR2 regulate Hepc *via* pathways involving both extracellular signal-regulated kinase (Erk) 1/2 and Smad1/5/8 [84]. HFE deficiency downregulates Bmp6/Smad signaling and induces iron overload [85, 86]. HFE expression in the retina is detected exclusively at the basolateral membrane in the RPE, suggesting that HFE mediates iron uptake from choroidal blood into the retina as it interacts with TfR1/2 [41].

### Regulation of ferritin expression

Retinal Ft is regulated by iron regulatory proteins (IRPs) [63]. IRP1 and IRP2 are two cytosolic proteins that modulate cellular iron homeostasis by binding to stem-loop structures called iron-responsive elements (IREs) in the untranslated regions (UTR) of their target mRNAs, which encode proteins involved in iron metabolism [87]. IRP1 and IRP2 are proteins share 56% sequence homology [87]. IRP2 has an additional cysteine-rich 73 amino acid insertion in its N-terminus with unknown function [88]. Both IRP1 and IRP2 are expressed ubiquitously [87] and cytosolic IRP1/2 modulates the labile iron pool. When cellular iron levels are low, IRP binds to the IREs either in the 5’ UTR of *Ft* mRNA or the 3’ UTR of *TfR1* and *DMT1* mRNA [53, 89]. The binding of IRPs to IREs on the 5’ UTR of *Ft* mRNA inhibits translation by blocking elongation. In contrast, the binding of IRPs to IREs on the 3′ UTR of *TfR1* and *DMT1* mRNA increases mRNA stability [53, 89]. While IRP2 functions solely as an RNA-binding protein, IRP1 operates as both an RNA-binding protein and a cytosolic aconitase [90]. When cellular iron levels are high, IRP1 binds to a [4Fe-4S] cluster and functions as an iron-sulfur aconitase to catalyze the conversion of citrate to isocitrate [89]. Under iron-deficient conditions, IRP1 loses its iron-sulfur cluster and acquires IRE-binding activity [91].

### Regulation of Fpn expression

The expression of the transmembrane iron exporter Fpn is regulated by Hepc at the post-translational level [46] *via* Hepc-mediated internalization and degradation [83]. Hepc is expressed in photoreceptors, Müller cells, and the RPE, suggesting that the retina may produce it for local iron regulation [92]. In the RPE, Fpn is expressed in the basolateral membrane [63]. Thus, Fpn may be regulated by Hepc in the systemic circulation [42]. In the inner retina, retina-derived Hepc may regulate Fpn independent of HFE [92]. Hepc upregulation and consequent Fpn downregulation have been associated with increased oxidative stress and apoptosis in the murine retina [92]. Hepc binding to Fpn recruits and activates Janus kinase 2 (Jak2), which in turn is required for Fpn phosphorylation [42, 93]. Moreover, α2-macroglobulin (α2M) binds Hepc in the circulation, potentially enhancing Hepc sequestration [94]. The α2M-Hepc complex is more effective in inducing Fpn degradation than Hepc alone [94]. Notably, Hepc expression is affected by multiple stimuli including iron level, erythropoiesis rate, inflammation, hypoxia, and oxidative stress [95], through cell surface proteins including HFE, TfR2, hemojuvelin (Hjv), matriptase2, and interleukin 6 (IL-6) [96]. The proteins induce Hepc expression by regulating its transcription [97], and activating various cell signal transduction pathways, including the bone morphogenic protein 6 (Bmp)/Smad, Janus kinase/signal transducers and activators of transcription (Jak/Stat) and hypoxia-inducible factor (Hif)-1 pathways [96]. A previous study suggested that the Erk pathway is responsible for Hepc upregulation in the retina [83]. However, other studies suggest that Bmp6 could upregulate Hepc in the retina, with the absence of Bmp6 or Hepc causing similar retinal iron accumulation in mice [98].

Hjv is an important iron-handling protein that modulates Fpn by regulating Hepc expression. Unlike HFE, Hjv can either be localized on the cell membrane through a glycosylphosphatidylinositol anchor or released in a soluble form [99, 100]. Membrane-anchored Hjv acts as a coreceptor for Bmp family proteins and induces Hepc expression [99, 101] through phosphorylation of Smads 1/5/8 [102, 103]. Soluble Hjv antagonizes and consequently blocks Bmp signaling and phosphorylation of Smads, thereby suppressing Hepc expression [101]. Matriptase-2, a serine protease also known as transmembrane protease serine 6 (TMPRSS6), is expressed in all retina cell types [97] and generates soluble Hjv [99]. It also suppresses Hepc transcription through proteolytic Hjv processing on the cell membrane [104]. Matriptase-2 is expressed on the apical membrane of the RPE and is also co-localized with Hjv [97]. The release of soluble Hjv is induced by the transmembrane receptor neogenin [101], and occurs after the Hjv-neogenin complex is internalized from the cell surface [105]. Bmp6 is unable to induce Hepc expression in Hjv-null RPE cells, confirming its role in Hjv-dependent induction of Hepc in the retina [98, 106]. Hjv is highly expressed in RPE, Mülller cells, photoreceptor cells, and retinal ganglion cells [107]. Additionally, Hjv expression in RPE is restricted to the apical membrane [97], indicating that Hepc expression in the RPE may be regulated by the HFE-Hjv complex whereas Hepc expression in the neural retina may be regulated by Hjv [108].

Amyloid-β (Aβ) precursor protein (APP) promotes iron export by binding to and stabilizing Fpn [109]. Increased hippocampal and cortical neuronal iron and oxidation have been observed in *App* null mutant mice [110]. In contrast, APP is regulated by iron with excessive intracellular iron levels causing the IRPs to dissociate from their IRE binding sites in *APP* mRNA [110, 111] and enhancing APP translation [112]. Chronic iron overload increases the processing of APP byproducts, generating toxic Aβ species in the RPE and drusen, heterogeneous debris external to the RPE, a hallmark of AMD [110, 111, 113].

## Iron accumulation and lipid peroxidation in the aging retina and amd pathogenesis

### Iron causes oxidative stress and lipid peroxidation in the retina

**Figure 3.**
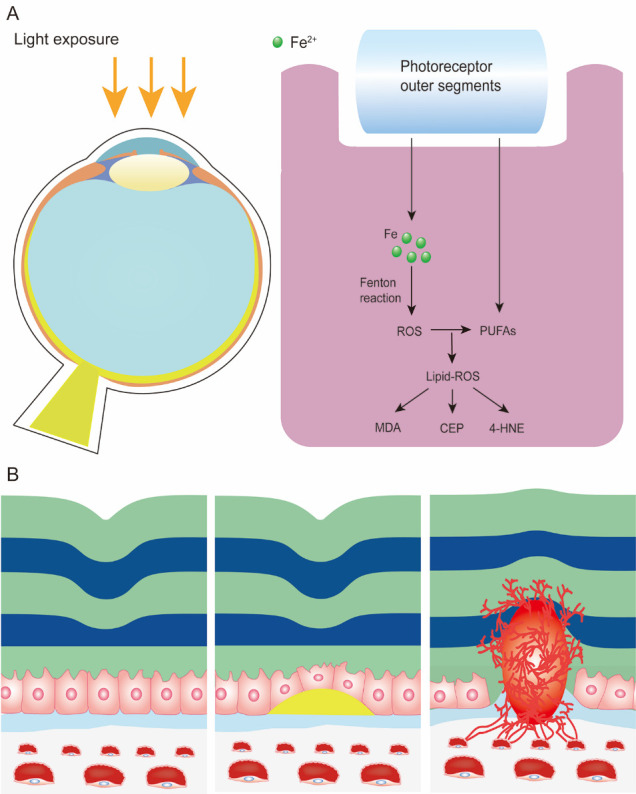
Iron accumulation and lipid peroxidation in the aging retina and AMD. (A) Life-long irradiation exposure causes constant phagocytosis of iron-laden and polyunsaturated fatty acid (PUFA)-enriched photoreceptor outer segments in the RPE. Reactive oxygen species (ROS) generated from accumulated iron in the RPE *via* Fenton reactions further reacts with PUFAs to generate lipid-ROS and promote lipid peroxidation. Products of lipid peroxidation, including carboxyethylpyrrole (CEP), 4-hydroxynonenal (4-HNE), and malondialdehyde (MDA), cause a series of inflammatory responses and AMD features. (B) Patterns of AMD. Left: Normal structure of the macula. Middle: Dry AMD, also known as non-exudative AMD. is characterised by heterogeneous debris (drusen) accumulation between the RPE and Bruch’s membrane. Right: Wet AMD (also known as exudative AMD) is characterised by choroidal neovascularization underneath the RPE and macula. Abnormal blood vessels may then break the continuity of RPE and Bruch’s membrane and cause sub-retinal hemorrhage.

Several iron-containing proteins are involved in the phototransduction cascade in the retina [42]. For example, the RPE65 protein is expressed in RPE and converts 11-cis-retinal to all-trans-retinyl as part of the retinoid cycle necessary for iron-dependent phototransduction [114]. Additionally, iron is indispensable for guanylate cyclase in synthesizing the second messenger cGMP in the phototransduction pathway [42]. However, excess iron can be toxic by forming ROS through the Fenton reaction, initiated by a reaction between ferrous iron and hydrogen peroxide [115] (Fig. 3A). The largest percentage of intracellular iron is tightly bound to or incorporated into proteins as a cofactor or for storage. However, a small portion (less than 1%) of intracellular iron (the labile iron pool) is localized in the cytosol and intracellular organelles including lysosomes and mitochondria [29, 35, 116, 117]. Under physiological conditions, iron is highly reactive and converts endogenously produced hydrogen peroxide to highly reactive intermediate species [118], including hydroxyl radicals (HO^•^) and high-valence oxo-ferryl species [26, 119]. The iron centers of lipoxygenases (LOXs) can also catalyze the formation of primary enzymatic products of lipid peroxidation, hydroperoxy lipids (L-OOH), whereas Fe^2+^ from the labile iron pool participates in secondary L-OOH decomposition reactions to produce oxidatively truncated electrophilic products of lipid peroxidation [120]. When iron levels exceed the cellular anti-oxidative capacity, aberrant ROS production damages DNA, proteins, and lipids within the retina [121, 122].

ROS reacts with PUFAs in plasma and membrane organelles that generate lipid peroxides [123]. In the disc membrane of retinal photoreceptors, the long-chain PUFA docosahexaenoic acid (DHA) contributes 50% of the total fatty acid content of phospholipids and accounts for 75-100% of fatty acids [124]. Notably, cell types with relatively high levels of PUFAs, such as retinal cells are highly sensitive to lipid peroxidation. Such sensitivity can be reduced by lipid antioxidants including vitamin E and GPX4 [125, 126]. Iron was initially reported to contribute to lipid peroxidation-associated pathological changes in murine retina in the early 1960s; this action could be prevented by vitamin E [127]. Two major lipid peroxidation products, malondialdehyde (MDA) and 4-hydroxynonenal (4-HNE), increase in the photoreceptor inner segments after intravenous iron injection [128]. Notably, photoreceptor cells exhibit heterogeneity in their susceptibilities to iron-mediated oxidative damage: cones are more susceptible than rods and other retinal cells [129]. This phenomenon may be due to the different components of the endogenous antioxidant defense system that provide varying levels of protection. Another possibility is that the cone discs are more accessible to extracellular iron due to the lack of an outer membrane surrounding them [130].

Exposure to visible light induces iron release from Ft, causing lipid peroxidation in the photoreceptor outer segment [131]. Light exposure renders the retina susceptible to oxidative stress-mediated damage, whereas iron overload increases oxidative stress and radiosensitivity [132]. DHA further aggravates photo-oxidative damage in RPE cells through lipid peroxidation [133]. It also increases susceptibility to photo-oxidation-induced retinal degeneration [134]. Microarray analysis of light damaged murine retina revealed changes in many iron regulatory genes, including upregulation of *Tf* and *Tfrc* and downregulation of *Hfe*, *Bmp6* and *Heph* in both the neurosensory retina and the RPE, with an overall iron-overload state and iron-induced oxidative stress [30]. Under photo-oxidative conditions, heme oxygenase 1 is upregulated and catalyzes the production of ferrous iron and carbon monoxide, which may exacerbate cellular injury and oxidative stress by generating free radicals [30], further enhancing the vulnerability of the retina to oxidative stress and suggesting a feed-forward cycle of photo-oxidative stress-induced damage [30]. High antioxidant content in the retina (e.g., GSH, vitamin E and C, and macular carotenoids) may counteract light-induced oxidative damage [135-137]. Interestingly, intracellular iron triggers the regulatory effect of glutamate synthesis and secretion in RPE cells through its effects on cytosolic aconitase activity [138-140]. Iron-induced GSH increase results in increased glutamate/cystine (Cys_2_) antiporter (system x_c_^-^) activity with increased levels of Cys_2_ uptake. This subsequently increases intracellular GSH levels [36, 106, 139]. HFE deficiency-induced iron overload triggers a compensatory system x_c_^-^ upregulation and GSH elevation to encounter iron-induced oxidative stress [141]. In the aging retina, antioxidant defense mechanisms have reduced activity, thereby increasing vulnerability to oxidative stress and iron-induced oxidative damage [142].

### Accumulated iron and elevated lipid peroxidation in AMD pathogenesis

Each RPE cell phagocytoses thousands of outer segment discs that are rich in iron and PUFAs [143], producing iron accumulation in the RPE [4, 83]. Unless exported, this accumulated iron either binds to melanin in RPE cells or accumulates to toxic levels [48]. Increased iron levels have been reported in the RPE, outer retina, and choroid in the elderly [4, 53, 144]. In comparison to younger persons, elderly subjects display an approximate 2-fold increase in iron levels in the body [145], a 3-fold increase in the RPE [146], and a 1.3-fold increase in the neuroretina [147]. Age-induced retinal iron accumulation is associated with alterations in iron-regulatory proteins including TfR, Ft, Fpn, and Cp [145, 146], with different changes observed in the RPE/choroid and the neuroretina [74, 146]. *FTL1* mRNA expression has been reported to be significantly increased in the aged retina, whereas *FTH1* mRNA expression was not changed with age [146]. Higher Hepc levels and lower TfR levels have been reported in aged *Bmp6* null mutant mice relative to age-matched wild-type (WT) mice, suggesting that increased Hepc levels are insufficient to prevent further iron influx [128]. Cp and Heph double knockout mice showed an age-dependent increase in iron levels in the RPE and retina, with AMD features, including age-dependent RPE hypertrophy, hyperplasia and death, photoreceptor degeneration, and subretinal neovascularization [74]. Of note, ferrosenescence, referred to as a vicious cycle between aging and iron accumulation has recently been reported [148]. Intracellular iron accumulation triggers genomic disintegration, promoting aging by inducing DNA damage, while blocking DNA repair and additional age-related iron retention [148]. Additionally, high PUFA content in the retina aggravates the photo-oxidation-induced senescence of RPE cells [133].

Residues from phagocytized photoreceptors tend to accumulate with age, forming lipofuscin granules in the RPE [149]. These granules are characterized by phototoxicity and contain carboxyethylpyrrole (CEP), an oxidation fragment of DHA, and fluorescent bisretinoid derivatives including N-retinylidene-N-retinylethanol-amine (A2E) [150]. CEP and A2E are toxic to RPE cells and eventually become part of drusen, contributing to degeneration of the RPE with loss of adherent photoreceptors thus representing a clinical hallmark of AMD [149]. Bisretinoid lipofuscin and iron can also initiate photooxidative damage. Therefore, iron chelation, either independently or in combination with bisretinoid inhibitors could potentially serve as treatments for AMD [151-153].

The retina is separated from the systemic circulation by the inner and outer BRBs established by the tight junctions between neighboring retinal endothelial cells and RPE cells respectively [154]. Therefore, it is believed that this tissue is immune to changes in systemic circulation [42]. Breakdown of the inner BRB results in the death of retinal endothelial cells under pathological conditions including oxidative stress, leukostasis, endothelial progenitor dysfunction, and senescence [155]. Lipid peroxidation in aged retinal vessels also leads to retinal endothelial cell death [156], which may lead to retinal iron accumulation, thus causing retinal degeneration [47, 155]. Previous studies have found that age-related changes in iron levels in the retina are local and are not associated with changes in blood iron levels [146]. Notably, dietary iron supplementation only modestly increased iron levels in the RPE of WT mice [157]. Another study demonstrated that a high systemic iron level resulted in mouse retinal iron accumulation despite an intact BRB [128]. The RPE is a component of the outer BRB. As a result, the BRB protects against the influx of iron from the serum to the inner retina but not to the RPE [128, 157, 158]. Long-term iron administration with aging alters retinal and choroid structures and the expression of iron-handling proteins [159]. Hepc produced locally in the retina is insufficient to prevent retinal iron uptake in cases of increased blood iron levels due to aging. Therefore, systemic iron levels significantly increase iron levels in the RPE and choroid [128], leading to histological features similar to those of AMD [158]. These results are consistent with observations in a clinical case employing long-term intravenous iron therapy [158].

Retinal iron accumulation is a characteristic feature of AMD. It contributes to AMD pathogenesis predominantly by inducing oxidative stress-mediated damage and inflammation [160]. Retinal iron accumulation due to aceruloplasminemia revealed AMD features, including RPE alteration and formation of drusen, lipofuscin, and melanolipofuscin granules [161]. In AMD, iron is present in the photoreceptors, RPE, and Bruch’s membrane, including drusen, the clinical hallmark of AMD [6, 160, 162, 163], with increased levels of iron-handling proteins Tf, Ft, and Fpn [31, 163, 164]. Intracellular iron is mainly stored in Ft; thus, changes in Ft alter intracellular iron content. Serum Ft is considered a marker for iron storage and an independent indicator of early AMD [165]. Mutations in the gene encoding mitochondrial Ft (FtMt), an iron-storage protein specifically localized in retinal mitochondria, may result in reduced protection from iron-dependent oxidative stress in the mitochondria, thus causing AMD pathogenesis [166]. An age-related increase in FtMt has been observed in murine RPE [167]. Increased FtMt potentially induces a biphasic response in aged RPE and AMD. In aged RPE under normoxic conditions, it may prevent macular degeneration by triggering mitophagy and enhancing antioxidant effect. However, hypoxic conditions caused by drusen accumulation between the RPE and Bruch’s membrane may decrease levels of mature FtMt in the RPE. This results in reduced protection against age-related stress, which may cause RPE degeneration and contribute to the pathogenesis of dry AMD [167]. Moreover, increased FtMt levels upregulate vascular endothelial growth factor (VEGF) secretion to induce choroidal neovascularization, the leading cause of wet AMD [167] (Fig. 3B). The combined deficiency of iron ferroxidases Heph and Cp in mice increases Ft levels and is associated with retinal degeneration due to iron overload and AMD-like features including drusen formation and subretinal neovascularization [74, 168]. The peptide hormone Hepc regulates iron content by preventing iron export by triggering degradation of the iron exporter Fpn [83]. The absence of Hepc likely allows increased iron uptake into the retina from the retinal vasculature through Fpn, which is localized in the vascular endothelium and exports iron from the abluminal side of these cells [98]. Therefore, a decrease in Hepc may lead to iron overload in the retina, with downregulation of Hepc implicated in AMD development. Hepc levels are significantly lower in the aqueous humor of patients with AMD than in control subjects [169]. The absence of Hepc results in age-dependent retinal iron accumulation, followed by retinal degeneration, in mice [83]. Since HFE is important for transcriptional regulation of Hepc, its absence also results in retinal iron accumulation similar to that observed in *Hepc* knockout mice [141]. Moreover, *DMT1* polymorphism may be a potential environment-dependent risk marker for AMD [170]. Collectively, these findings suggest that altered iron metabolism plays an important role in AMD pathogenesis.

**Figure 4.**
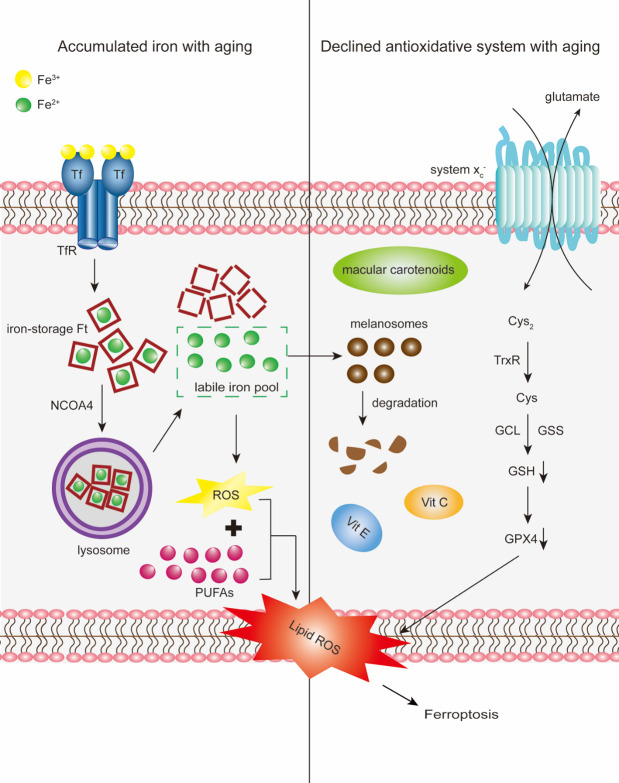
Schematic of the proposed involvement of ferroptosis in the aging retina and AMD. Cargo receptor NCOA4 delivers iron-storage macromolecule Ft to the lysosomes, where Ft is then degraded. Fe^2+^released into the cytoplasm from degraded Ft constitutes the labile iron pool. With aging, accumulated iron exceeds the storage capacity of retinal cells, enters the labile iron pool and expands the redox-active iron pool. Fe^2+^ from the labile iron pool subsequently generates ROS *via* Fenton reactions. ROS further react with PUFAs to generate lipid-ROS and promote lipid peroxidation. Conversely, a decline in lipid antioxidants with aging, particularly GSH, alters the antioxidative capacity of retinal cells. Synthesis of GSH from glutamate, cysteine (Cys), and glycine occurs *via* two steps catalyzed by two ATP-dependent cytoplasmic enzymes namely glutamate-cysteine ligase (GCL) and glutathione synthetase (GSS). Intracellular GSH biosynthesis is dependent on the availability of Cys, the reduced form of cystine (Cys_2_) catalyzed by thioredoxin reductase (TrxR). Cys_2_ uptake is mainly mediated by the system x_c_^-^, the upstream determinant of ferroptosis. Decline in GSH with aging ultimately inactivates GPX4, the sole enzyme that reduces lipid hydroperoxides within biological membranes. Iron-mediated melanosome degradation reduces its ability to inhibit iron-induced lipid peroxidation. Iron accumulation and decline in lipid antioxidants, coordinatively aggravate age-related iron-induced lipid peroxidation, initiating ferroptosis.

Iron-induced oxidative stress plays a role in the pathophysiology of AMD [160, 163, 164]. The retina is one of the highest oxygen-consuming tissues in the human body [171]. High oxygen consumption, constant light exposure, enriched PUFAs, and the presence of photosensitizers increase ROS production in the retina [172, 173]. Oxidative-stress-induced ROS during aging overwhelms the antioxidative capability, causing modification of and damage to the retina [13]. Notably, antioxidant defense mechanisms that scavenge ROS are essential for redox homeostasis in the retina [174]. However, in the aged retina, normal antioxidant defense mechanisms gradually become inefficient, thereby predisposing to oxidative stress [142]. In addition, iron-mediated degradation of melanosomes reduces their ability to inhibit iron-induced lipid peroxidation [161, 175] (Fig 4). Age-related increases in lipofuscin, 8-oxoguanine, CEP, 4-HNE, and MDA expression have been observed in the aging retina [176-179], which have been reported to cause inflammatory responses and AMD features [180, 181]. Within the retina, the macular is more susceptible to lipid peroxidation than the mid-peripheral retina, which is reflected by immunoreactivity to 4-HNE [177, 179].

Excessive iron is proangiogenic [62]. Iron mediates succinate receptor-G-protein-coupled receptor 91 (GPR91) signaling in retinal ganglion and RPE cells, and stimulates the expression and secretion of VEGF [108]. Deletion of *Hjv* results in iron overload in murine retina and consequently causes abnormal retinal angiogenesis, which is reminiscent of the AMD process [182]. Iron is also involved in the inflammatory aspect of AMD pathogenesis. For example, light-induced free radicals form 7-ketocholesterol, a highly toxic cholesterol oxide that utilizes iron to induce inflammation and is associated with AMD [183, 184]. The Fenton reaction is the predominant process whereby 7-ketocholesterol is formed in the retina [185]. Iron reportedly upregulates complement C3 transcription and protein activation [186]. Intravenous iron injection may increase the complement deposition on the basal side of the RPE [158]. Subretinal iron injection also causes inflammasome-mediated toxicity in the RPE [187]. Additionally, iron overload may downregulate the cholesterol efflux transporters-ATP binding cassette subfamily A member 1 (ABCA1) and ATP-binding cassette subfamily G member 1 (ABCG1) with a concurrent increase in retinal cholesterol content. Since excessive cholesterol is pro-inflammatory, iron overload may promote retinal inflammation *via* cholesterol in AMD [188]. Furthermore, it has been reported that iron-induced retinal toxicity requires the NLRP3 inflammasome [187], an immune signaling complex that has been implicated in AMD pathogenesis [189-194].

## Effect of iron accumulation and lipid peroxidation on ferroptosis

Ferroptosis is initiated by lipid peroxidation due to compromised GSH-dependent antioxidant systems, such as GSH downregulation/depletion or GPX4 inhibition [18]. It is characterized by iron accumulation and lipid peroxidation, and morphologically distinct from other forms of regulated cell death [21, 195]. It is generally accepted that free intracellular iron catalyzes lipid peroxidation during ferroptosis [27].

High levels of intracellular iron are prerequisites for initiating ferroptosis. Ferroptotic cell death, whether induced by Cys_2_ deprivation, system x_c_^-^ inhibition, or direct GPX4 inhibition, can be suppressed by iron chelators, knockdown of the expression of iron transporter Tf and its receptor TfR, or exclusion of iron in serum [20, 21, 196-198]. Similarly, inhibiting iron exposure to lipoxygenases can drive ferroptosis through PUFA peroxidation [199]. In contrast, the addition of iron or iron-bound Tf to the growth medium has been shown to accelerate erastin-induced ferroptosis [21, 200]. Iron supplementation has been shown to enhance ferroptotic death in mice defective in system x_c_^-^ [201]. Since intracellular free iron exists as part of the labile iron pool, changes in iron uptake, storage, or export modify the labile iron pool content and intracellular redox-active iron level [202]. Mechanistically, intracellular iron induces ROS elevation *via* Fenton reactions which in turn reacts with PUFAs to form lipid peroxides [203]. Such Fenton reaction-mediated propagation of lipid peroxidation ultimately results in ferroptosis [204] (Fig 4).

Iron that initiates ferroptosis may either be imported or released through a process termed ferritinophagy, in which the iron storage macromolecule Ft is degraded *via* autophagy [198, 205, 206]. Ferritinophagy is mediated by cargo receptor nuclear receptor coactivator 4 (NCOA4), which delivers Ft to lysosomes or through heme catabolism [196, 205, 207-210]. *NCOA4* knockdown has been reported to prevent erastin-induced ferroptosis [206]. In ferritinophagy, autophagy leads to iron-dependent ferroptosis by degradation of cellular iron stock protein Ft and induction of TfR1 expression [198, 206]. Degradation of Ft increases cellular labile iron levels, thereby ensuring rapid accumulation of cellular ROS and ultimately ferroptosis [206]. In autophagy-deficient cells, such as *BECN1*^+/-^ and *LC3B*^-/-^ cells, cellular labile iron and ROS levels remain unchanged due to compromised autophagy. Thus, ferroptosis inducers cannot initiate ferroptosis in autophagy-deficient cells [211]. Deficiency of lysosome-associated membrane protein-2 (LAMP2), a highly glycosylated protein involved in chaperone-mediated autophagy, may reduce cytosolic cysteine (Cys) concentration, resulting in low GSH and causing ferroptosis [212]. Ft degradation increases cellular labile iron levels to initiate ferroptosis [206]. Induction of expression the iron-carrier protein TfR, which mediates iron import is required in ferroptosis [200]. Cells exhibiting altered iron hemostasis, such as genetic ablation of iron uptake (*Tf* and *TfR1*), metabolism (*IRP2*), and storage (*FTH1*) genes are resistant to ferroptosis [21, 196, 200, 213]. Moreover, ferroptosis can be prevented by iron chelators [21, 196, 209]. The iron chelator deferoxamine is internalized through endocytosis and accumulates in lysosomes, suggesting that it can prevent ferroptosis by chelating the labile iron in the lysosomes [213]. Mitochondrial iron chaperon frataxin-deficient adipocytes are susceptible to ferroptosis and exhibit increased lipid peroxidation and decreased GPX4 expression [214]. Frataxin selectively recognizes Fe^2+^, which is utilized by diverse Fe^2+^-dependent proteins. Therefore, iron may play multiple roles in ferroptosis independent of its redox activity, by modulating the activities of iron-containing proteins [215, 216].

Lipid peroxidation is a key initiator of ferroptosis [217]. Iron enhances the production of lipid peroxides through iron-dependent oxidases such as lipoxygenases [2], non-heme iron-containing enzymes that can catalyze deoxygenation of PUFAs in lipids thereby generating the proximate inducers of ferroptosis [215]. The downstream intervention pathways of lipid peroxidation include both GPX4-dependent and -independent pathways. GPX4 is the sole enzyme that reduces lipid hydroperoxides to lipid alcohols (LOOH) within biological membranes. Its integral role in mitigating lipid peroxidation makes it a critical anti-ferroptotic mediator [197, 218, 219]. Consistently, the loss of GPX4 function, along with increased free iron availability and oxidation of PUFAs, have recently been proposed as hallmarks of ferroptosis [220]. Inhibiting system x_c_^-^ (a type 1 ferroptosis inducer) suppresses Cys_2_/glutamate exchange and downregulates intracellular GSH levels, which directly inactivates GPX4 (a type 2 ferroptosis inducer) [18].

In addition to thiol-dependent antioxidative systems, cellular lipophilic antioxidants inhibit lipid peroxidation and ferroptosis due to radical-trapping [6]. Recent studies indicate that ferroptosis can be prevented by GSH/GPX4-independent systems. A recent study showed that ferroptosis-suppressing protein 1 (FSP1), also known as apoptosis-inducing factor mitochondrial 2 (AIFM2), is a key regenerator of endogenous CoQ_10_ in the membrane; inhibition of FSP1, in contrast, activates the ferroptotic cascade in *GPX4*-null environments [221, 222]. The FSP1 myristoylation-binding motif recruits itto the plasma membrane, where it generates radical-trapping antioxidants to counteract lipid peroxidation, and eventually prevents ferroptosis [27]. In most situations, the FSP1/CoQ_10_ protection branch is secondary to GPX4, but could become primary upon increased metabolic needs. Many more questions need to be answered before clinical applications can be developed [223].

## Potential involvement of ferroptosis in AMD pathogenesis

AMD pathogenesis is linked to oxidative stress-induced cell death in the RPE and subsequent death of the overlying photoreceptors [29]. However, the nature of RPE/photoreceptor cell death in AMD is controversial. Earlier studies suggested that apoptosis is the major mechanism for RPE/photoreceptor cell death in AMD [13]. More recent studies have uncovered important contributions from other mechanisms, including pyroptosis, necroptosis, autophagy, and ferroptosis [13-17, 29, 212, 224]. Notably, ferroptosis is important in the pathogenesis of ischemia-reperfusion injury and neurodegenerative diseases [225]. Elevated brain iron levels caused by dietary iron supplementation contribute to ferroptosis-induced neuronal degeneration and loss [112]. Inducing ferroptosis in mice leads to features that resemble Alzheimer’s disease, including neuronal loss and astrogliosis in the hippocampus [226]. In contrast, administering a ferroptosis inhibitor could prevent the progression of several neurodegenerative diseases, including Huntington's disease, Parkinson's disease, Alzheimer’s disease, and periventricular leukomalacia [227-231].

Several reports support ferroptotic cell death in AMD. The presence of iron in the culture medium with low glutamate concentration (causes intracellular GSH depletion) induced retinal ganglion cell death [146]. GPX4 knockdown has also been reported to induce lipid peroxidation and retinal cell death [126, 232]. In contrast, GPX4 overexpression suppresses VEGF and reduces the size of choroidal neovascularization, a hallmark of late-stage AMD [233]. Due to technological limitations, investigators were unable to clearly identify ferroptosis. Moreover, emerging evidence has shown that exogenous stimuli such as *tert*-butyl hydroperoxide and GSH depletion-induced lipid peroxidation alter the expression of genes associated with iron metabolism [29, 224]. This results in a feed-forward cycle of ROS elevation and iron accumulation that ultimately induces ferroptosis in RPE cells [29, 224]. The Age-Related Eye Disease Study demonstrated that dietary supplementation of nutrients with lipid antioxidant properties (e.g., lutein and zeaxanthin, zinc, vitamin C and E, and β-carotene) can reduce the risk of AMD progression [234, 235]. Age-associated decline in the efficiency of retinal redox systems predisposes the retina to increased oxidative stress-mediated damage and promotes AMD progression [236, 237]. GSH depletion can inactivate GPX4 and consequently induces ferroptosis [29]. A significantly reduced potential of the antioxidant defense system, including GPX, has been reported in AMD patients and animal models [238, 239]. LAMP2 deficiency, which contributes to the formation of RPE basolaminar deposits and AMD progression [240], increases the risk of ROS-induced ferroptosis in RPE cells [212].

The protective effect of iron chelators against AMD supports the contribution of ferroptosis to AMD [83, 241-243]. Iron chelators were initially designed to treat iron-overload diseases [244]. Recent studies have reported the effect of iron chelation therapy in retinal degeneration and in several neurodegenerative disorders, including Alzheimer's disease, Parkinson's disease, and amyotrophic lateral sclerosis [2, 245].

Current clinically available iron chelators include deferoxamine (Desferal), deferiprone (Ferriprox), deferasirox (Exjade), and salicylaldehyde isonicotinoyl hydrazine (SIH) [246]. The most effective of these is deferoxamine [247], naturally secreted by the bacterium *Streptomyces pilosus* [244]. As a membrane-impermeable iron chelator, it accumulates in the lysosome through endocytosis [248], suggesting that it prevents ferroptosis by chelating labile iron in lysosomes [213]. Deferoxamine can also bind to iron, thereby preventing iron-generated ROS [244]. Therefore, it is efficient in chelation therapy by alleviating symptoms of oxidative damage evident in iron-overload diseases [249]. However, the potential of deferoxamine as a therapeutic agent is limited by its route of administration, short plasma half-life, high hydrophilicity, severe retinal toxicity [250-252], bone dysplasia, and auditory toxicity [253]. Ocular side effects of deferoxamine include cataract, optic neuropathy, optic atrophy, and macular or equatorial pigmentary degeneration [254]. The retinal toxicity of deferoxamine primarily targets the RPE-Bruch membrane-photoreceptor complex that extends from the peri-fovea to the peripheral retina and is associated with foveola sparing [251], and alters retinal function [250, 251, 255]. Iron pro-chelators have been developed that bind to iron only when activated by oxidative stress [256]. Zinc deferoxamine, a zinc complex of deferoxamine, has also been developed to enhance cellular permeability [257]; a single intraperitoneal injection induces deferoxamine accumulation in the retina, which attenuates retinal degeneration and oxidative stress-induced excitoneurotoxicity without significant retinal toxicity [258, 259].

In comparison to deferoxamine, deferiprone and deferasirox have relatively low molecular weight (139.15 and 373 g/mol, respectively) [260, 261]. They are orally active and have longer half-lives than deferoxamine [262]. More rapid chelation has been observed with deferiprone and deferasirox relative to deferoxamine in several cultured cell types [263]. Deferiprone and deferasirox can cross membranes including the blood-brain barrier and BRB, readily [259], thus decreasing retinal labile iron levels [241] and attenuating retinopathy caused by multiple stimuli-induced oxidative stresses and lipid peroxidation [259, 264]. Reports of the toxicities of deferiprone and deferasirox have been controversial [265, 266]. Deferiprone is less toxic than deferoxamine [267]; however, deferasirox was reported to have severe side effects, including fatal renal/hepatic impairment or failure, and gastrointestinal hemorrhage [268]. The ocular toxicity associated with deferoxamine has not been reported with deferiprone [254]. Notably, long-term administration of deferiprone decreases iron levels and oxidative stress in the retina and RPE without ocular toxicity [242]. However, it has been reported that deferasirox administration may cause toxic maculopathy, a type of retinopathy [269]. Deferiprone protects against light-induced [270] and tunicamycin-induced retinal photoreceptor degeneration through its inhibitory effect on endoplasmic reticulum stress [245]. Deferasirox protects retinal neurons against excitoneurotoxicity by reducing iron content and oxidative stress *in vivo* [259].

SIH is a highly lipophilic and cell-permeable iron chelator [271-273] that protects RPE cells against oxidative stress and cell death induced by multiple stimuli [243, 274, 275]. Alternative mechanisms underlying its protective action may include activation of the Nrf2 transcription factor, which in turn regulates the expression of antioxidant genes [276].

Systemic administration of α-lipoic acid (an antioxidant and iron chelator) protects against light-induced photoreceptor degeneration in the murine retina [277]. Systemic administration or local delivery of the iron transport protein Tf, an endogenous iron chelator, attenuates iron accumulation in the retina and protects against retinal degeneration in a light-induced and AMD mouse model [278, 279].

## Conclusion

Life-long phagocytosis of photoreceptor outer segments causes iron accumulation and lipid peroxidation with aging [83, 143], which are characteristic features of AMD [6, 160, 162, 163]. Specific components of ferroptosis implicated in AMD pathogenesis include ROS production, iron accumulation, and lipid peroxidation. Defective lipid repair systems predispose the aging retina to increased oxidative stress-mediated damage [280] and ferroptosis [29].

Ferroptosis was implicated in retinal cell death before the cell death processes were discovered [126, 146, 232, 233, 281-283]. However, the reported mechanisms were attributed to other cell-death phenotypes, such as oxytosis, a type of cell death that conforms to the same pathway as ferroptosis [232, 284]. Recent studies have demonstrated that exogenous stimuli-induced lipid peroxidation induces ferroptosis in RPE cells [29, 224]. The first report that directly links ferroptosis and oxidative stress due to compromised antioxidant defense mechanisms in the retina has been published and suggests a potential role for ferroptosis in AMD [29, 224]. Furthermore, photooxidative damage results in ROS elevation, causing oxidative stress-induced RPE/photoreceptor cell death and contributing to AMD pathogenesis [133, 280]. Several other types of regulated cell death, such as apoptosis [134, 285] and pyroptosis [14], have also been reported [286]. Novel agents targeting ferroptosis as potential treatments for AMD include iron chelators, lipophilic antioxidants, and inhibitors of lipid peroxidation [18]; the characteristic features have been observed in AMD patients and animal models [234, 235].

Conclusively, several current studies link ferroptosis with the aged retina and AMD. Further studies are required to determine the mechanisms of cell death in AMD and to identify the precise relationships between ferroptosis, the aged retina, and AMD.

## References

[1] Hirayama T (2019). Fluorescent probes for the detection of catalytic Fe(II) ion. Free Radic Biol Med, 133:38-45. 29990536 10.1016/j.freeradbiomed.2018.07.004

[2] Masaldan S, Bush AI, Devos D, Rolland AS, Moreau C (2019). Striking while the iron is hot: Iron metabolism and ferroptosis in neurodegeneration. Free Radic Biol Med, 133:221-233. 30266679 10.1016/j.freeradbiomed.2018.09.033

[3] Andrews NC (1999). Disorders of iron metabolism. N Engl J Med, 341:1986-1995. 10607817 10.1056/NEJM199912233412607

[4] Ugarte M, Geraki K, Jeffery G (2018). Aging results in iron accumulations in the non-human primate choroid of the eye without an associated increase in zinc, copper or sulphur. Biometals, 31:1061-1073. 30306383 10.1007/s10534-018-0147-x PMC6245114

[5] Belaidi AA, Bush AI (2016). Iron neurochemistry in Alzheimer's disease and Parkinson's disease: targets for therapeutics. J Neurochem, 139 Suppl 1:179-197. 10.1111/jnc.13425 26545340

[6] Biesemeier A, Yoeruek E, Eibl O, Schraermeyer U (2015). Iron accumulation in Bruch's membrane and melanosomes of donor eyes with age-related macular degeneration. Exp Eye Res, 137:39-49. 26026877 10.1016/j.exer.2015.05.019

[7] de Jong PT (2006). Age-related macular degeneration. N Engl J Med, 355:1474-1485. 17021323 10.1056/NEJMra062326

[8] Cai X, McGinnis JF (2012). Oxidative stress: the achilles' heel of neurodegenerative diseases of the retina. Front Biosci (Landmark Ed), 17:1976-1995. 22201850 10.2741/4033

[9] Datta S, Cano M, Ebrahimi K, Wang L, Handa JT (2017). The impact of oxidative stress and inflammation on RPE degeneration in non-neovascular AMD. Prog Retin Eye Res, 60:201-218. 28336424 10.1016/j.preteyeres.2017.03.002 PMC5600827

[10] Bridges CC, Kekuda R, Wang H, Prasad PD, Mehta P, Huang W, et al. (2001). Structure, function, and regulation of human cystine/glutamate transporter in retinal pigment epithelial cells. Invest Ophthalmol Vis Sci, 42:47-54. 11133847

[11] Njie-Mbye YF, Kulkarni-Chitnis M, Opere CA, Barrett A, Ohia SE (2013). Lipid peroxidation: pathophysiological and pharmacological implications in the eye. Front Physiol, 4:366. 24379787 10.3389/fphys.2013.00366 PMC3863722

[12] Adler R, Curcio C, Hicks D, Price D, Wong F (1999). Cell death in age-related macular degeneration. Mol Vis, 5:31. 10562655

[13] Hanus J, Anderson C, Wang S (2015). RPE necroptosis in response to oxidative stress and in AMD. Ageing Res Rev, 24:286-298. 26369358 10.1016/j.arr.2015.09.002 PMC4661094

[14] Wooff Y, Fernando N, Wong JHC, Dietrich C, Aggio-Bruce R, Chu-Tan JA, et al. (2020). Caspase-1-dependent inflammasomes mediate photoreceptor cell death in photo-oxidative damage-induced retinal degeneration. Sci Rep, 10:2263. 32041990 10.1038/s41598-020-58849-z PMC7010818

[15] Ma H, Yang F, Ding XQ (2020). Inhibition of thyroid hormone signaling protects retinal pigment epithelium and photoreceptors from cell death in a mouse model of age-related macular degeneration. Cell Death Dis, 11:24. 31932580 10.1038/s41419-019-2216-7 PMC6957507

[16] Yumnamcha T, Devi TS, Singh LP (2019). Auranofin mediates mitochondrial dysregulation and inflammatory cell death in human retinal pigment epithelial cells: implications of retinal neurodegenerative diseases. Front Neurosci, 13:1065. 31649499 10.3389/fnins.2019.01065 PMC6795687

[17] Kaarniranta K, Tokarz P, Koskela A, Paterno J, Blasiak J (2017). Autophagy regulates death of retinal pigment epithelium cells in age-related macular degeneration. Cell Biol Toxicol, 33:113-128. 27900566 10.1007/s10565-016-9371-8 PMC5325845

[18] Stockwell BR, Friedmann Angeli JP, Bayir H, Bush AI, Conrad M, Dixon SJ, et al. (2017). Ferroptosis: A regulated cell death nexus linking metabolism, redox biology, and disease. Cell, 171:273-285. 28985560 10.1016/j.cell.2017.09.021 PMC5685180

[19] Yang WS, Stockwell BR (2016). Ferroptosis: death by lipid peroxidation. Trends Cell Biol, 26:165-176. 26653790 10.1016/j.tcb.2015.10.014 PMC4764384

[20] Dixon SJ, Stockwell BR (2014). The role of iron and reactive oxygen species in cell death. Nat Chem Biol, 10:9-17. 24346035 10.1038/nchembio.1416

[21] Dixon SJ, Lemberg KM, Lamprecht MR, Skouta R, Zaitsev EM, Gleason CE, et al. (2012). Ferroptosis: an iron-dependent form of nonapoptotic cell death. Cell, 149:1060-1072. 22632970 10.1016/j.cell.2012.03.042 PMC3367386

[22] Galluzzi L, Vitale I, Aaronson SA, Abrams JM, Adam D, Agostinis P, et al. (2018). Molecular mechanisms of cell death: recommendations of the Nomenclature Committee on Cell Death 2018. Cell Death Differ, 25:486-541. 29362479 10.1038/s41418-017-0012-4 PMC5864239

[23] Friedmann Angeli JP, Schneider M, Proneth B, Tyurina YY, Tyurin VA, Hammond VJ, et al. (2014). Inactivation of the ferroptosis regulator Gpx4 triggers acute renal failure in mice. Nat Cell Biol, 16:1180-1191. 25402683 10.1038/ncb3064 PMC4894846

[24] Anandhan A, Dodson M, Schmidlin CJ, Liu P, Zhang DD (2020). Breakdown of an Ironclad Defense System: The Critical Role of NRF2 in Mediating Ferroptosis. Cell Chem Biol, 27:436-447. 32275864 10.1016/j.chembiol.2020.03.011 PMC7597851

[25] Tonnus W, Gembardt F, Latk M, Parmentier S, Hugo C, Bornstein SR, et al. (2019). The clinical relevance of necroinflammation-highlighting the importance of acute kidney injury and the adrenal glands. Cell Death Differ, 26:68-82. 30224638 10.1038/s41418-018-0193-5 PMC6294800

[26] Bayir H, Anthonymuthu TS, Tyurina YY, Patel SJ, Amoscato AA, Lamade AM, et al. (2020). Achieving life through death: redox biology of lipid peroxidation in ferroptosis. Cell Chem Biol, 27:387-408. 32275865 10.1016/j.chembiol.2020.03.014 PMC7218794

[27] Belavgeni A, Meyer C, Stumpf J, Hugo C, Linkermann A (2020). Ferroptosis and necroptosis in the kidney. Cell Chem Biol, 27:448-462. 32302582 10.1016/j.chembiol.2020.03.016

[28] Green DR (2019). The coming decade of cell death research: five riddles. Cell, 177:1094-1107. 31100266 10.1016/j.cell.2019.04.024 PMC6534278

[29] Sun Y, Zheng Y, Wang C, Liu Y (2018). Glutathione depletion induces ferroptosis, autophagy, and premature cell senescence in retinal pigment epithelial cells. Cell Death Dis, 9:753. 29988039 10.1038/s41419-018-0794-4 PMC6037763

[30] Hadziahmetovic M, Kumar U, Song Y, Grieco S, Song D, Li Y, et al. (2012). Microarray analysis of murine retinal light damage reveals changes in iron regulatory, complement, and antioxidant genes in the neurosensory retina and isolated RPE. Invest Ophthalmol Vis Sci, 53:5231-5241. 22736611 10.1167/iovs.12-10204 PMC4159963

[31] Chowers I, Wong R, Dentchev T, Farkas RH, Iacovelli J, Gunatilaka TL, et al. (2006). The iron carrier transferrin is upregulated in retinas from patients with age-related macular degeneration. Invest Ophthalmol Vis Sci, 47:2135-2140. 16639025 10.1167/iovs.05-1135

[32] Chua SYL, Khawaja AP, Dick AD, Morgan J, Dhillon B, Lotery AJ, et al. (2020). Ambient air pollution associations with retinal morphology in the UK biobank. Invest Ophthalmol Vis Sci, 61:32. 10.1167/iovs.61.5.32 PMC7405693 32428233

[33] Theil EC, Goss DJ (2009). Living with iron (and oxygen): questions and answers about iron homeostasis. Chem Rev, 109:4568-4579. 19824701 10.1021/cr900052g PMC2919049

[34] Ganz T (2013). Systemic iron homeostasis. Physiol Rev, 93:1721-1741. 24137020 10.1152/physrev.00008.2013

[35] Wang J, Pantopoulos K (2011). Regulation of cellular iron metabolism. Biochem J, 434:365-381. 21348856 10.1042/BJ20101825 PMC3048577

[36] Harned J, Ferrell J, Nagar S, Goralska M, Fleisher LN, McGahan MC (2012). Ceruloplasmin alters intracellular iron regulated proteins and pathways: ferritin, transferrin receptor, glutamate and hypoxia-inducible factor-1alpha. Exp Eye Res, 97:90-97. 22343016 10.1016/j.exer.2012.02.001 PMC3535017

[37] Puri C (2009). Loss of myosin VI no insert isoform (NoI) induces a defect in clathrin-mediated endocytosis and leads to caveolar endocytosis of transferrin receptor. J Biol Chem, 284:34998-35014. 19840950 10.1074/jbc.M109.012328 PMC2787362

[38] Yefimova MG, Jeanny JC, Guillonneau X, Keller N, Nguyen-Legros J, Sergeant C, et al. (2000). Iron, ferritin, transferrin, and transferrin receptor in the adult rat retina. Invest Ophthalmol Vis Sci, 41:2343-2351. 10892882

[39] Muckenthaler MU, Rivella S, Hentze MW, Galy B (2017). A red carpet for iron metabolism. Cell, 168:344-361. 28129536 10.1016/j.cell.2016.12.034 PMC5706455

[40] Kawabata H, Yang R, Hirama T, Vuong PT, Kawano S, Gombart AF, et al. (1999). Molecular cloning of transferrin receptor 2. A new member of the transferrin receptor-like family. J Biol Chem, 274:20826-20832. 10409623 10.1074/jbc.274.30.20826

[41] Martin PM, Gnana-Prakasam JP, Roon P, Smith RG, Smith SB, Ganapathy V (2006). Expression and polarized localization of the hemochromatosis gene product HFE in retinal pigment epithelium. Invest Ophthalmol Vis Sci, 47:4238-4244. 17003411 10.1167/iovs.06-0026

[42] Gnana-Prakasam JP, Martin PM, Smith SB, Ganapathy V (2010). Expression and function of iron-regulatory proteins in retina. IUBMB Life, 62:363-370. 20408179 10.1002/iub.326 PMC3789380

[43] Hunt RC, Dewey A, Davis AA (1989). Transferrin receptors on the surfaces of retinal pigment epithelial cells are associated with the cytoskeleton. J Cell Sci, 92(Pt 4):655-666. 2600139 10.1242/jcs.92.4.655

[44] Burdo JR, Antonetti DA, Wolpert EB, Connor JR (2003). Mechanisms and regulation of transferrin and iron transport in a model blood-brain barrier system. Neuroscience, 121:883-890. 14580938 10.1016/s0306-4522(03)00590-6

[45] Sterling J, Guttha S, Song Y, Song D, Hadziahmetovic M, Dunaief JL (2017). Iron importers Zip8 and Zip14 are expressed in retina and regulated by retinal iron levels. Exp Eye Res, 155:15-23. 28057442 10.1016/j.exer.2016.12.008 PMC5359041

[46] Theurl M, Song D, Clark E, Sterling J, Grieco S, Altamura S, et al. (2016). Mice with hepcidin-resistant ferroportin accumulate iron in the retina. FASEB J, 30:813-823. 26506980 10.1096/fj.15-276758 PMC4714557

[47] Baumann B, Sterling J, Song Y, Song D, Fruttiger M, Gillies M, et al. (2017). Conditional muller cell ablation leads to retinal iron accumulation. Invest Ophthalmol Vis Sci, 58:4223-4234. 28846772 10.1167/iovs.17-21743 PMC5574447

[48] Baksi S, Tripathi AK, Singh N (2016). Alpha-synuclein modulates retinal iron homeostasis by facilitating the uptake of transferrin-bound iron: Implications for visual manifestations of Parkinson's disease. Free Radic Biol Med, 97:292-306. 27343690 10.1016/j.freeradbiomed.2016.06.025 PMC4996775

[49] Steere AN, Byrne SL, Chasteen ND, Mason AB (2012). Kinetics of iron release from transferrin bound to the transferrin receptor at endosomal pH. Biochim Biophys Acta, 1820:326-333. 21699959 10.1016/j.bbagen.2011.06.003 PMC3253137

[50] McKie AT (2005). A ferrireductase fills the gap in the transferrin cycle. Nat Genet, 37:1159-1160. 16254556 10.1038/ng1105-1159

[51] Mayle KM, Le AM, Kamei DT (2012). The intracellular trafficking pathway of transferrin. Biochim Biophys Acta, 1820:264-281. 21968002 10.1016/j.bbagen.2011.09.009 PMC3288267

[52] Ohgami RS, Campagna DR, Greer EL, Antiochos B, McDonald A, Chen J, et al. (2005). Identification of a ferrireductase required for efficient transferrin-dependent iron uptake in erythroid cells. Nat Genet, 37:1264-1269. 16227996 10.1038/ng1658 PMC2156108

[53] He X, Hahn P, Iacovelli J, Wong R, King C, Bhisitkul R, et al. (2007). Iron homeostasis and toxicity in retinal degeneration. Prog Retin Eye Res, 26:649-673. 17921041 10.1016/j.preteyeres.2007.07.004 PMC2093950

[54] Kidane TZ, Sauble E, Linder MC (2006). Release of iron from ferritin requires lysosomal activity. Am J Physiol Cell Physiol, 291:C445-455. 16611735 10.1152/ajpcell.00505.2005

[55] Kerins MJ, Ooi A (2017). The roles of NRF2 in modulating cellular iron homeostasis. Antioxid Redox Signal, 29:1756-1773. 28793787 10.1089/ars.2017.7176 PMC6208163

[56] Truman-Rosentsvit M, Berenbaum D, Spektor L, Cohen LA, Belizowsky-Moshe S, Lifshitz L, et al. (2018). Ferritin is secreted via 2 distinct nonclassical vesicular pathways. Blood, 131:342-352. 29074498 10.1182/blood-2017-02-768580 PMC5774206

[57] Cohen LA, Gutierrez L, Weiss A, Leichtmann-Bardoogo Y, Zhang DL, Crooks DR, et al. (2010). Serum ferritin is derived primarily from macrophages through a nonclassical secretory pathway. Blood, 116:1574-1584. 20472835 10.1182/blood-2009-11-253815

[58] Baumann BH, Shu W, Song Y, Simpson EM, Lakhal-Littleton S, Dunaief JL (2019). Ferroportin-mediated iron export from vascular endothelial cells in retina and brain. Exp Eye Res, 187:107728. 31323276 10.1016/j.exer.2019.107728 PMC6759385

[59] Mendes-Jorge L, Ramos D, Valenca A, Lopez-Luppo M, Pires VM, Catita J, et al. (2014). L-ferritin binding to scara5: a new iron traffic pathway potentially implicated in retinopathy. PLoS One, 9:e106974. 25259650 10.1371/journal.pone.0106974 PMC4178024

[60] Li JY, Paragas N, Ned RM, Qiu A, Viltard M, Leete T, et al. (2009). Scara5 is a ferritin receptor mediating non-transferrin iron delivery. Dev Cell, 16:35-46. 19154717 10.1016/j.devcel.2008.12.002 PMC2652503

[61] Goralska M, Holley BL, McGahan MC (2001). Overexpression of H- and L-ferritin subunits in lens epithelial cells: Fe metabolism and cellular response to UVB irradiation. Invest Ophthalmol Vis Sci, 42:1721-1727. 11431434

[62] Coffman LG, Parsonage D, D'Agostino R Jr, Torti FM, Torti SV (2009). Regulatory effects of ferritin on angiogenesis. Proc Natl Acad Sci U S A, 106:570-575. 19126685 10.1073/pnas.0812010106 PMC2626744

[63] Hahn P, Dentchev T, Qian Y, Rouault T, Harris ZL, Dunaief JL (2004). Immunolocalization and regulation of iron handling proteins ferritin and ferroportin in the retina. Mol Vis, 10:598-607. 15354085

[64] Silva B, Faustino P (2015). An overview of molecular basis of iron metabolism regulation and the associated pathologies. Biochim Biophys Acta, 1852:1347-1359. 25843914 10.1016/j.bbadis.2015.03.011

[65] Kruszewski M (2003). Labile iron pool: the main determinant of cellular response to oxidative stress. Mutat Res, 531:81-92. 14637247 10.1016/j.mrfmmm.2003.08.004

[66] Bulteau AL, O'Neill HA, Kennedy MC, Ikeda-Saito M, Isaya G, Szweda LI (2004). Frataxin acts as an iron chaperone protein to modulate mitochondrial aconitase activity. Science, 305:242-245. 15247478 10.1126/science.1098991

[67] Shi H, Bencze KZ, Stemmler TL, Philpott CC (2008). A cytosolic iron chaperone that delivers iron to ferritin. Science, 320:1207-1210. 18511687 10.1126/science.1157643 PMC2505357

[68] Yanatori I, Richardson DR, Imada K, Kishi F (2016). Iron export through the transporter ferroportin 1 is modulated by the iron chaperone PCBP2. J Biol Chem, 291:17303-17318. 27302059 10.1074/jbc.M116.721936 PMC5016129

[69] Rice AE, Mendez MJ, Hokanson CA, Rees DC, Bjorkman PJ (2009). Investigation of the biophysical and cell biological properties of ferroportin, a multipass integral membrane protein iron exporter. J Mol Biol, 386:717-732. 19150361 10.1016/j.jmb.2008.12.063 PMC2677177

[70] Gunshin H, Mackenzie B, Berger UV, Gunshin Y, Romero MF, Boron WF, et al. (1997). Cloning and characterization of a mammalian proton-coupled metal-ion transporter. Nature, 388:482-488. 9242408 10.1038/41343

[71] Yanatori I, Yasui Y, Tabuchi M, Kishi F (2014). Chaperone protein involved in transmembrane transport of iron. Biochem J, 462:25-37. 24854545 10.1042/BJ20140225

[72] Donovan A, Brownlie A, Zhou Y, Shepard J, Pratt SJ, Moynihan J, et al. (2000). Positional cloning of zebrafish ferroportin1 identifies a conserved vertebrate iron exporter. Nature, 403:776-781. 10693807 10.1038/35001596

[73] Burkhart A, Skjorringe T, Johnsen KB, Siupka P, Thomsen LB, Nielsen MS, et al. (2016). Expression of iron-related proteins at the neurovascular unit supports reduction and reoxidation of iron for transport through the blood-brain barrier. Mol Neurobiol, 53:7237-7253. 26687231 10.1007/s12035-015-9582-7

[74] Hahn P, Qian Y, Dentchev T, Chen L, Beard J, Harris ZL, et al. (2004). Disruption of ceruloplasmin and hephaestin in mice causes retinal iron overload and retinal degeneration with features of age-related macular degeneration. Proc Natl Acad Sci U S A, 101:13850-13855. 15365174 10.1073/pnas.0405146101 PMC518844

[75] Wilkinson N, Pantopoulos K (2013). IRP1 regulates erythropoiesis and systemic iron homeostasis by controlling HIF2alpha mRNA translation. Blood, 122:1658-1668. 23777768 10.1182/blood-2013-03-492454

[76] Kautz L, Jung G, Valore EV, Rivella S, Nemeth E, Ganz T (2014). Identification of erythroferrone as an erythroid regulator of iron metabolism. Nat Genet, 46:678-684. 24880340 10.1038/ng.2996 PMC4104984

[77] Shah YM, Xie L (2014). Hypoxia-inducible factors link iron homeostasis and erythropoiesis. Gastroenterology, 146:630-642. 24389303 10.1053/j.gastro.2013.12.031 PMC3943938

[78] Maxwell PH, Ferguson DJ, Nicholls LG, Iredale JP, Pugh CW, Johnson MH, et al. (1997). Sites of erythropoietin production. Kidney Int, 51:393-401. 9027712 10.1038/ki.1997.52

[79] Testi C, Boffi A, Montemiglio LC (2019). Structural analysis of the transferrin receptor multifaceted ligand(s) interface. Biophys Chem, 254:106242. 31419721 10.1016/j.bpc.2019.106242

[80] Giannetti AM, Bjorkman PJ (2004). HFE and transferrin directly compete for transferrin receptor in solution and at the cell surface. J Biol Chem, 279:25866-25875. 15056661 10.1074/jbc.M401467200

[81] Wessling-Resnick M (2018). Crossing the iron gate: why and how transferrin receptors mediate viral entry. Annu Rev Nutr, 38:431-458. 29852086 10.1146/annurev-nutr-082117-051749 PMC6743070

[82] Gao J, Chen J, Kramer M, Tsukamoto H, Zhang AS, Enns CA (2009). Interaction of the hereditary hemochromatosis protein HFE with transferrin receptor 2 is required for transferrin-induced hepcidin expression. Cell Metab, 9:217-227. 19254567 10.1016/j.cmet.2009.01.010 PMC2673483

[83] Hadziahmetovic M, Song Y, Ponnuru P, Iacovelli J, Hunter A, Haddad N, et al. (2011). Age-dependent retinal iron accumulation and degeneration in hepcidin knockout mice. Invest Ophthalmol Vis Sci, 52:109-118. 20811044 10.1167/iovs.10-6113 PMC3053271

[84] Wallace DF, Summerville L, Crampton EM, Frazer DM, Anderson GJ, Subramaniam VN (2009). Combined deletion of Hfe and transferrin receptor 2 in mice leads to marked dysregulation of hepcidin and iron overload. Hepatology, 50:1992-2000. 19824072 10.1002/hep.23198

[85] Kautz L, Meynard D, Besson-Fournier C, Darnaud V, Al Saati T, Coppin H, et al. (2009). BMP/Smad signaling is not enhanced in Hfe-deficient mice despite increased Bmp6 expression. Blood, 114:2515-2520. 19622835 10.1182/blood-2009-02-206771

[86] Corradini E, Garuti C, Montosi G, Ventura P, Andriopoulos B Jr, Lin HY, et al. (2009). Bone morphogenetic protein signaling is impaired in an HFE knockout mouse model of hemochromatosis. Gastroenterology, 137:1489-1497. 19591830 10.1053/j.gastro.2009.06.057 PMC2757523

[87] Ghosh MC, Zhang DL, Rouault TA (2015). Iron misregulation and neurodegenerative disease in mouse models that lack iron regulatory proteins. Neurobiol Dis, 81:66-75. 25771171 10.1016/j.nbd.2015.02.026 PMC4567523

[88] Bourdon E, Kang DK, Ghosh MC, Drake SK, Wey J, Levine RL, et al. (2003). The role of endogenous heme synthesis and degradation domain cysteines in cellular iron-dependent degradation of IRP2. Blood Cells Mol Dis, 31:247-255. 12972033 10.1016/s1079-9796(03)00161-x

[89] Hirota K (2019). An intimate crosstalk between iron homeostasis and oxygen metabolism regulated by the hypoxia-inducible factors (HIFs). Free Radic Biol Med, 133:118-129. 30053508 10.1016/j.freeradbiomed.2018.07.018

[90] Wilkinson N, Pantopoulos K (2014). The IRP/IRE system in vivo: insights from mouse models. Front Pharmacol, 5:176. 25120486 10.3389/fphar.2014.00176 PMC4112806

[91] Zhang DL, Ghosh MC, Rouault TA (2014). The physiological functions of iron regulatory proteins in iron homeostasis - an update. Front Pharmacol, 5:124. 24982634 10.3389/fphar.2014.00124 PMC4056636

[92] Gnana-Prakasam JP, Martin PM, Mysona BA, Roon P, Smith SB, Ganapathy V (2008). Hepcidin expression in mouse retina and its regulation via lipopolysaccharide/Toll-like receptor-4 pathway independent of Hfe. Biochem J, 411:79-88. 18042040 10.1042/BJ20071377 PMC3731152

[93] De Domenico I, Lo E, Ward DM, Kaplan J (2009). Hepcidin-induced internalization of ferroportin requires binding and cooperative interaction with Jak2. Proc Natl Acad Sci U S A, 106:3800-3805. 19234114 10.1073/pnas.0900453106 PMC2656160

[94] Peslova G, Petrak J, Kuzelova K, Hrdy I, Halada P, Kuchel PW, et al. (2009). Hepcidin, the hormone of iron metabolism, is bound specifically to alpha-2-macroglobulin in blood. Blood, 113:6225-6236. 19380872 10.1182/blood-2009-01-201590

[95] Ganz T (2006). Hepcidin and its role in regulating systemic iron metabolism. Hematology Am Soc Hematol Educ Program:29-35, 507. 10.1182/asheducation-2006.1.29 17124036

[96] Darshan D, Anderson GJ (2009). Interacting signals in the control of hepcidin expression. Biometals, 22:77-87. 19130266 10.1007/s10534-008-9187-y

[97] Gnana-Prakasam JP, Baldowski RB, Ananth S, Martin PM, Smith SB, Ganapathy V (2014). Retinal expression of the serine protease matriptase-2 (Tmprss6) and its role in retinal iron homeostasis. Mol Vis, 20:561-574. 24791141 PMC4000719

[98] Hadziahmetovic M, Song Y, Wolkow N, Iacovelli J, Kautz L, Roth MP, et al. (2011). Bmp6 regulates retinal iron homeostasis and has altered expression in age-related macular degeneration. Am J Pathol, 179:335-348. 21703414 10.1016/j.ajpath.2011.03.033 PMC3123855

[99] Arjunan P, Gnanaprakasam JP, Ananth S, Romej MA, Rajalakshmi VK, Prasad PD, et al. (2016). Increased retinal expression of the pro-angiogenic receptor GPR91 via BMP6 in a mouse model of juvenile hemochromatosis. Invest Ophthalmol Vis Sci, 57:1612-1619. 27046124 10.1167/iovs.15-17437 PMC4824383

[100] Lin L, Goldberg YP, Ganz T (2005). Competitive regulation of hepcidin mRNA by soluble and cell-associated hemojuvelin. Blood, 106:2884-2889. 15998830 10.1182/blood-2005-05-1845

[101] Xia Y, Babitt JL, Sidis Y, Chung RT, Lin HY (2008). Hemojuvelin regulates hepcidin expression via a selective subset of BMP ligands and receptors independently of neogenin. Blood, 111:5195-5204. 18326817 10.1182/blood-2007-09-111567 PMC2384142

[102] Wang RH, Li C, Xu X, Zheng Y, Xiao C, Zerfas P, et al. (2005). A role of SMAD4 in iron metabolism through the positive regulation of hepcidin expression. Cell Metab, 2:399-409. 16330325 10.1016/j.cmet.2005.10.010

[103] Babitt JL, Huang FW, Wrighting DM, Xia Y, Sidis Y, Samad TA, et al. (2006). Bone morphogenetic protein signaling by hemojuvelin regulates hepcidin expression. Nat Genet, 38:531-539. 16604073 10.1038/ng1777

[104] Ramsay AJ, Hooper JD, Folgueras AR, Velasco G, Lopez-Otin C (2009). Matriptase-2 (TMPRSS6): a proteolytic regulator of iron homeostasis. Haematologica, 94:840-849. 19377077 10.3324/haematol.2008.001867 PMC2688576

[105] Zhang AS, Yang F, Meyer K, Hernandez C, Chapman-Arvedson T, Bjorkman PJ, et al. (2008). Neogenin-mediated hemojuvelin shedding occurs after hemojuvelin traffics to the plasma membrane. J Biol Chem, 283:17494-17502. 18445598 10.1074/jbc.M710527200 PMC2427329

[106] Gnana-Prakasam JP, Tawfik A, Romej M, Ananth S, Martin PM, Smith SB, et al. (2012). Iron-mediated retinal degeneration in haemojuvelin-knockout mice. Biochem J, 441:599-608. 21943374 10.1042/BJ20111148 PMC3710445

[107] Ramey G, Deschemin JC, Vaulont S (2009). Cross-talk between the mitogen activated protein kinase and bone morphogenetic protein/hemojuvelin pathways is required for the induction of hepcidin by holotransferrin in primary mouse hepatocytes. Haematologica, 94:765-772. 19454495 10.3324/haematol.2008.003541 PMC2688567

[108] Gnana-Prakasam JP, Ananth S, Prasad PD, Zhang M, Atherton SS, Martin PM, et al. (2011). Expression and iron-dependent regulation of succinate receptor GPR91 in retinal pigment epithelium. Invest Ophthalmol Vis Sci, 52:3751-3758. 21357408 10.1167/iovs.10-6722 PMC3109050

[109] Wong BX, Tsatsanis A, Lim LQ, Adlard PA, Bush AI, Duce JA (2014). beta-Amyloid precursor protein does not possess ferroxidase activity but does stabilize the cell surface ferrous iron exporter ferroportin. PLoS One, 9:e114174. 25464026 10.1371/journal.pone.0114174 PMC4252103

[110] Duce JA, Tsatsanis A, Cater MA, James SA, Robb E, Wikhe K, et al. (2010). Iron-export ferroxidase activity of beta-amyloid precursor protein is inhibited by zinc in Alzheimer's disease. Cell, 142:857-867. 20817278 10.1016/j.cell.2010.08.014 PMC2943017

[111] Guo LY, Alekseev O, Li Y, Song Y, Dunaief JL (2014). Iron increases APP translation and amyloid-beta production in the retina. Exp Eye Res, 129:31-37. 25456519 10.1016/j.exer.2014.10.012 PMC4259833

[112] Li LB, Chai R, Zhang S, Xu SF, Zhang YH, Li HL, et al. (2019). Iron exposure and the cellular mechanisms linked to neuron degeneration in adult mice. Cells, 8. 10.3390/cells8020198 PMC6406573 30813496

[113] Dasari B, Prasanthi JR, Marwarha G, Singh BB, Ghribi O (2010). The oxysterol 27-hydroxycholesterol increases beta-amyloid and oxidative stress in retinal pigment epithelial cells. BMC Ophthalmol, 10:22. 20836858 10.1186/1471-2415-10-22 PMC2946278

[114] Moiseyev G, Chen Y, Takahashi Y, Wu BX, Ma JX (2005). RPE65 is the isomerohydrolase in the retinoid visual cycle. Proc Natl Acad Sci U S A, 102:12413-12418. 16116091 10.1073/pnas.0503460102 PMC1194921

[115] Enami S, Sakamoto Y, Colussi AJ (2014). Fenton chemistry at aqueous interfaces. Proc Natl Acad Sci U S A, 111:623-628. 24379389 10.1073/pnas.1314885111 PMC3896178

[116] Ward DM, Cloonan SM (2019). Mitochondrial iron in human health and disease. Annu Rev Physiol, 81:453-482. 30485761 10.1146/annurev-physiol-020518-114742 PMC6641538

[117] Seo AY, Xu J, Servais S, Hofer T, Marzetti E, Wohlgemuth SE, et al. (2008). Mitochondrial iron accumulation with age and functional consequences. Aging Cell, 7:706-716. 18843794 10.1111/j.1474-9726.2008.00418.x PMC3849824

[118] Galaris D, Barbouti A, Pantopoulos K (2019). Iron homeostasis and oxidative stress: An intimate relationship. Biochim Biophys Acta Mol Cell Res, 1866:118535. 31446062 10.1016/j.bbamcr.2019.118535

[119] Yamamoto N, Koga N, Nagaoka M (2012). Ferryl-oxo species produced from Fenton's reagent via a two-step pathway: minimum free-energy path analysis. J Phys Chem B, 116:14178-14182. 23148728 10.1021/jp310008z

[120] Stoyanovsky DA, Tyurina YY, Shrivastava I, Bahar I, Tyurin VA, Protchenko O, et al. (2019). Iron catalysis of lipid peroxidation in ferroptosis: Regulated enzymatic or random free radical reaction? Free Radic Biol Med, 133:153-161. 30217775 10.1016/j.freeradbiomed.2018.09.008 PMC6555767

[121] Hentze MW, Muckenthaler MU, Galy B, Camaschella C (2010). Two to tango: regulation of Mammalian iron metabolism. Cell, 142:24-38. 20603012 10.1016/j.cell.2010.06.028

[122] Torti SV, Torti FM (2013). Iron and cancer: more ore to be mined. Nat Rev Cancer, 13:342-355. 23594855 10.1038/nrc3495 PMC4036554

[123] Apel K, Hirt H (2004). Reactive oxygen species: metabolism, oxidative stress, and signal transduction. Annu Rev Plant Biol, 55:373-399. 15377225 10.1146/annurev.arplant.55.031903.141701

[124] Johansson I, Monsen VT, Pettersen K, Mildenberger J, Misund K, Kaarniranta K, et al. (2015). The marine n-3 PUFA DHA evokes cytoprotection against oxidative stress and protein misfolding by inducing autophagy and NFE2L2 in human retinal pigment epithelial cells. Autophagy, 11:1636-1651. 26237736 10.1080/15548627.2015.1061170 PMC4590664

[125] Hirschhorn T, Stockwell BR (2019). The development of the concept of ferroptosis. Free Radic Biol Med, 133:130-143. 30268886 10.1016/j.freeradbiomed.2018.09.043 PMC6368883

[126] Ueta T, Inoue T, Furukawa T, Tamaki Y, Nakagawa Y, Imai H, et al. (2012). Glutathione peroxidase 4 is required for maturation of photoreceptor cells. J Biol Chem, 287:7675-7682. 22207760 10.1074/jbc.M111.335174 PMC3293550

[127] Golberg L, Martin LE, Batchelor A (1962). Biochemical changes in the tissues of animals injected with iron. 3. Lipid peroxidation. Biochem J, 83:291-298. 13899654 10.1042/bj0830291 PMC1243547

[128] Zhao L, Li Y, Song D, Song Y, Theurl M, Wang C, et al. (2014). A high serum iron level causes mouse retinal iron accumulation despite an intact blood-retinal barrier. Am J Pathol, 184:2862-2867. 25174877 10.1016/j.ajpath.2014.07.008 PMC4215029

[129] Rogers BS, Symons RC, Komeima K, Shen J, Xiao W, Swaim ME, et al. (2007). Differential sensitivity of cones to iron-mediated oxidative damage. Invest Ophthalmol Vis Sci, 48:438-445. 17197565 10.1167/iovs.06-0528

[130] Shu W, Baumann BH, Song Y, Liu Y, Wu X, Dunaief JL (2020). Ferrous but not ferric iron sulfate kills photoreceptors and induces photoreceptor-dependent RPE autofluorescence. Redox Biol, 34:101469. 32362442 10.1016/j.redox.2020.101469 PMC7327978

[131] Ohishi K, Zhang XM, Moriwaki S, Hiramitsu T, Matsugo S (2006). In the presence of ferritin, visible light induces lipid peroxidation of the porcine photoreceptor outer segment. Free Radic Res, 40:799-807. 17015258 10.1080/10715760600555027

[132] Theriot CA, Westby CM, Morgan JLL, Zwart SR, Zanello SB (2016). High dietary iron increases oxidative stress and radiosensitivity in the rat retina and vasculature after exposure to fractionated gamma radiation. NPJ Microgravity, 2:16014. 28725729 10.1038/npjmgrav.2016.14 PMC5515516

[133] Liu Y, Zhang D, Wu Y, Ji B (2014). Docosahexaenoic acid aggravates photooxidative damage in retinal pigment epithelial cells via lipid peroxidation. J Photochem Photobiol B, 140:85-93. 25108204 10.1016/j.jphotobiol.2014.07.016

[134] Tanito M, Brush RS, Elliott MH, Wicker LD, Henry KR, Anderson RE (2009). High levels of retinal membrane docosahexaenoic acid increase susceptibility to stress-induced degeneration. J Lipid Res, 50:807-819. 19023138 10.1194/jlr.M800170-JLR200 PMC2666167

[135] Abokyi S, To CH, Lam TT, Tse DY (2020). Central role of oxidative stress in age-related macular degeneration: evidence from a review of the molecular mechanisms and animal models. Oxid Med Cell Longev, 2020:7901270. 32104539 10.1155/2020/7901270 PMC7035553

[136] Rozanowska M, Edge R, Land EJ, Navaratnam S, Sarna T, Truscott TG (2019). Scavenging of retinoid cation radicals by urate, trolox, and alpha-, beta-, gamma-, and delta-tocopherols. Int J Mol Sci, 20, 2799. 10.3390/ijms20112799 PMC6600601 31181693

[137] E BD, Marfany G (2020). The relevance of oxidative stress in the pathogenesis and therapy of retinal dystrophies. Antioxidants (Basel), 9:347. 10.3390/antiox9040347 PMC7222416 32340220

[138] McGahan MC, Harned J, Mukunnemkeril M, Goralska M, Fleisher L, Ferrell JB (2005). Iron alters glutamate secretion by regulating cytosolic aconitase activity. Am J Physiol Cell Physiol, 288:C1117-1124. 15613494 10.1152/ajpcell.00444.2004

[139] Lall MM, Ferrell J, Nagar S, Fleisher LN, McGahan MC (2008). Iron regulates L-cystine uptake and glutathione levels in lens epithelial and retinal pigment epithelial cells by its effect on cytosolic aconitase. Invest Ophthalmol Vis Sci, 49:310-319. 18172108 10.1167/iovs.07-1041

[140] Harned J, Nagar S, McGahan MC (2014). Hypoxia controls iron metabolism and glutamate secretion in retinal pigmented epithelial cells. Biochim Biophys Acta, 1840:3138-3144. 24972165 10.1016/j.bbagen.2014.06.012

[141] Gnana-Prakasam JP, Thangaraju M, Liu K, Ha Y, Martin PM, Smith SB, et al. (2009). Absence of iron-regulatory protein Hfe results in hyperproliferation of retinal pigment epithelium: role of cystine/glutamate exchanger. Biochem J, 424:243-252. 19715555 10.1042/BJ20090424 PMC3719389

[142] Finkel T, Holbrook NJ (2000). Oxidants, oxidative stress and the biology of ageing. Nature, 408:239-247. 11089981 10.1038/35041687

[143] Yefimova MG, Jeanny JC, Keller N, Sergeant C, Guillonneau X, Beaumont C, et al. (2002). Impaired retinal iron homeostasis associated with defective phagocytosis in Royal College of Surgeons rats. Invest Ophthalmol Vis Sci, 43:537-545. 11818402

[144] Hahn P, Ying GS, Beard J, Dunaief JL (2006). Iron levels in human retina: sex difference and increase with age. Neuroreport, 17:1803-1806. 17164668 10.1097/WNR.0b013e3280107776

[145] Sharon D, Blackshaw S, Cepko CL, Dryja TP (2002). Profile of the genes expressed in the human peripheral retina, macula, and retinal pigment epithelium determined through serial analysis of gene expression (SAGE). Proc Natl Acad Sci U S A, 99:315-320. 11756676 10.1073/pnas.012582799 PMC117558

[146] Chen H, Liu B, Lukas TJ, Suyeoka G, Wu G, Neufeld AH (2009). Changes in iron-regulatory proteins in the aged rodent neural retina. Neurobiol Aging, 30:1865-1876. 18308429 10.1016/j.neurobiolaging.2008.01.002 PMC2789556

[147] Chen H, Lukas TJ, Du N, Suyeoka G, Neufeld AH (2009). Dysfunction of the retinal pigment epithelium with age: increased iron decreases phagocytosis and lysosomal activity. Invest Ophthalmol Vis Sci, 50:1895-1902. 19151392 10.1167/iovs.08-2850

[148] Sfera A, Bullock K, Price A, Inderias L, Osorio C (2018). Ferrosenescence: The iron age of neurodegeneration? Mech Ageing Dev, 174:63-75. 29180225 10.1016/j.mad.2017.11.012

[149] Garcia-Castineiras S (2010). Iron, the retina and the lens: a focused review. Exp Eye Res, 90:664-678. 20230820 10.1016/j.exer.2010.03.003 PMC2919496

[150] Ng KP, Gugiu B, Renganathan K, Davies MW, Gu X, Crabb JS, et al. (2008). Retinal pigment epithelium lipofuscin proteomics. Mol Cell Proteomics, 7:1397-1405. 18436525 10.1074/mcp.M700525-MCP200 PMC2493379

[151] Sparrow JR (2016). Vitamin A-aldehyde adducts: AMD risk and targeted therapeutics. Proc Natl Acad Sci U S A, 113:4564-4569. 27071115 10.1073/pnas.1600474113 PMC4855581

[152] Ueda K, Kim HJ, Zhao J, Song Y, Dunaief JL, Sparrow JR (2018). Iron promotes oxidative cell death caused by bisretinoids of retina. Proc Natl Acad Sci U S A, 115:4963-4968. 29686088 10.1073/pnas.1722601115 PMC5948992

[153] Fagali N, Catala A (2009). Fe2+ and Fe3+ initiated peroxidation of sonicated and non-sonicated liposomes made of retinal lipids in different aqueous media. Chem Phys Lipids, 159:88-94. 19477315 10.1016/j.chemphyslip.2009.03.001

[154] Cunha-Vaz J (2017). The blood-retinal barrier in the management of retinal disease: EURETINA Award Lecture. Ophthalmologica, 237:1-10. 28152535 10.1159/000455809

[155] Klaassen I, Van Noorden CJ, Schlingemann RO (2013). Molecular basis of the inner blood-retinal barrier and its breakdown in diabetic macular edema and other pathological conditions. Prog Retin Eye Res, 34:19-48. 23416119 10.1016/j.preteyeres.2013.02.001

[156] Nag TC, Maurya M, Roy TS (2019). Age-related changes of the human retinal vessels: Possible involvement of lipid peroxidation. Ann Anat, 226:35-47. 31330304 10.1016/j.aanat.2019.06.007

[157] Bhoiwala DL, Song Y, Cwanger A, Clark E, Zhao LL, Wang C, et al. (2015). CD1 mouse retina is shielded from iron overload caused by a high iron diet. Invest Ophthalmol Vis Sci, 56:5344-5352. 26275132 10.1167/iovs.15-17026 PMC4539129

[158] Song D, Kanu LN, Li Y, Kelly KL, Bhuyan RK, Aleman T, et al. (2016). AMD-like retinopathy associated with intravenous iron. Exp Eye Res, 151:122-133. 27565570 10.1016/j.exer.2016.08.008 PMC5045814

[159] Kumar P, Nag TC, Jha KA, Dey SK, Kathpalia P, Maurya M, et al. (2017). Experimental oral iron administration: Histological investigations and expressions of iron handling proteins in rat retina with aging. Toxicology, 392:22-31. 28993186 10.1016/j.tox.2017.10.005

[160] Wong RW, Richa DC, Hahn P, Green WR, Dunaief JL (2007). Iron toxicity as a potential factor in AMD. Retina, 27:997-1003. 18040235 10.1097/IAE.0b013e318074c290

[161] Wolkow N, Song Y, Wu TD, Qian J, Guerquin-Kern JL, Dunaief JL (2011). Aceruloplasminemia: retinal histopathologic manifestations and iron-mediated melanosome degradation. Arch Ophthalmol, 129:1466-1474. 22084216 10.1001/archophthalmol.2011.309 PMC4089978

[162] Dunaief JL (2006). Iron induced oxidative damage as a potential factor in age-related macular degeneration: the Cogan Lecture. Invest Ophthalmol Vis Sci, 47:4660-4664. 17065470 10.1167/iovs.06-0568

[163] Hahn P, Milam AH, Dunaief JL (2003). Maculas affected by age-related macular degeneration contain increased chelatable iron in the retinal pigment epithelium and Bruch's membrane. Arch Ophthalmol, 121:1099-1105. 12912686 10.1001/archopht.121.8.1099

[164] Dentchev T, Hahn P, Dunaief JL (2005). Strong labeling for iron and the iron-handling proteins ferritin and ferroportin in the photoreceptor layer in age-related macular degeneration. Arch Ophthalmol, 123:1745-1746. 16344450 10.1001/archopht.123.12.1745

[165] Oh IH, Choi EY, Park JS, Lee CH (2016). Association of serum ferritin and kidney function with age-related macular degeneration in the general population. PLoS One, 11:e0153624. 27096155 10.1371/journal.pone.0153624 PMC4838228

[166] Stenirri S, Santambrogio P, Setaccioli M, Erba BG, Pia Manitto M, Rovida E, et al. (2012). Study of FTMT and ABCA4 genes in a patient affected by age-related macular degeneration: identification and analysis of new mutations. Clin Chem Lab Med, 50:1021-1029. 22706241 10.1515/cclm-2011-0854

[167] Wang X, Yang H, Yanagisawa D, Bellier JP, Morino K, Zhao S, et al. (2016). Mitochondrial ferritin affects mitochondria by stabilizing HIF-1alpha in retinal pigment epithelium: implications for the pathophysiology of age-related macular degeneration. Neurobiol Aging, 47:168-179. 27599360 10.1016/j.neurobiolaging.2016.07.025

[168] Hadziahmetovic M, Dentchev T, Song Y, Haddad N, He X, Hahn P, et al. (2008). Ceruloplasmin/hephaestin knockout mice model morphologic and molecular features of AMD. Invest Ophthalmol Vis Sci, 49:2728-2736. 18326691 10.1167/iovs.07-1472 PMC2569891

[169] Chen L, Ma B, Liu X, Hao Y, Yang X, Liu M (2020). H2 O2 induces oxidative stress damage through the BMP-6/SMAD/hepcidin axis. Dev Growth Differ, 62:139-146. 32012242 10.1111/dgd.12650

[170] Wysokinski D, Zaras M, Dorecka M, Waszczyk M, Szaflik J, Blasiak J, et al. (2012). An association between environmental factors and the IVS4+44C>A polymorphism of the DMT1 gene in age-related macular degeneration. Graefes Arch Clin Exp Ophthalmol, 250:1057-1065. 22371024 10.1007/s00417-012-1966-z PMC3382657

[171] Yu DY, Cringle SJ (2005). Retinal degeneration and local oxygen metabolism. Exp Eye Res, 80:745-751. 15939030 10.1016/j.exer.2005.01.018

[172] Beatty S, Koh H, Phil M, Henson D, Boulton M (2000). The role of oxidative stress in the pathogenesis of age-related macular degeneration. Surv Ophthalmol, 45:115-134. 11033038 10.1016/s0039-6257(00)00140-5

[173] Khandhadia S, Lotery A (2010). Oxidation and age-related macular degeneration: insights from molecular biology. Expert Rev Mol Med, 12:e34. 20959033 10.1017/S146239941000164X

[174] Sacca SC, Cutolo CA, Ferrari D, Corazza P, Traverso CE (2018). The eye, oxidative damage and polyunsaturated fatty acids. Nutrients, 10. 10.3390/nu10060668 PMC6024720 29795004

[175] Zadlo A, Rozanowska MB, Burke JM, Sarna TJ (2007). Photobleaching of retinal pigment epithelium melanosomes reduces their ability to inhibit iron-induced peroxidation of lipids. Pigment Cell Res, 20:52-60. 17250548 10.1111/j.1600-0749.2006.00350.x

[176] Jarrett SG, Boulton ME (2012). Consequences of oxidative stress in age-related macular degeneration. Mol Aspects Med, 33:399-417. 22510306 10.1016/j.mam.2012.03.009 PMC3392472

[177] Nag TC, Kumar P, Wadhwa S (2017). Age related distribution of 4-hydroxy 2-nonenal immunoreactivity in human retina. Exp Eye Res, 165:125-135. 28986146 10.1016/j.exer.2017.09.014

[178] Ethen CM, Reilly C, Feng X, Olsen TW, Ferrington DA (2007). Age-related macular degeneration and retinal protein modification by 4-hydroxy-2-nonenal. Invest Ophthalmol Vis Sci, 48:3469-3479. 17652714 10.1167/iovs.06-1058

[179] Nag TC, Wadhwa S, Alladi PA, Sanyal T (2011). Localization of 4-hydroxy 2-nonenal immunoreactivity in aging human retinal Muller cells. Ann Anat, 193:205-210. 21454059 10.1016/j.aanat.2011.02.004

[180] Hollyfield JG, Bonilha VL, Rayborn ME, Yang X, Shadrach KG, Lu L, et al. (2008). Oxidative damage-induced inflammation initiates age-related macular degeneration. Nat Med, 14:194-198. 18223656 10.1038/nm1709 PMC2748836

[181] Kapphahn RJ, Giwa BM, Berg KM, Roehrich H, Feng X, Olsen TW, et al. (2006). Retinal proteins modified by 4-hydroxynonenal: identification of molecular targets. Exp Eye Res, 83:165-175. 16530755 10.1016/j.exer.2005.11.017

[182] Tawfik A, Gnana-Prakasam JP, Smith SB, Ganapathy V (2014). Deletion of hemojuvelin, an iron-regulatory protein, in mice results in abnormal angiogenesis and vasculogenesis in retina along with reactive gliosis. Invest Ophthalmol Vis Sci, 55:3616-3625. 24812553 10.1167/iovs.13-13677 PMC4053073

[183] Pariente A, Pelaez R, Perez-Sala A, Larrayoz IM (2019). Inflammatory and cell death mechanisms induced by 7-ketocholesterol in the retina. Implications for age-related macular degeneration. Exp Eye Res, 187:107746. 31394101 10.1016/j.exer.2019.107746

[184] Larrayoz IM, Huang JD, Lee JW, Pascual I, Rodriguez IR (2010). 7-ketocholesterol-induced inflammation: involvement of multiple kinase signaling pathways via NFkappaB but independently of reactive oxygen species formation. Invest Ophthalmol Vis Sci, 51:4942-4955. 20554621 10.1167/iovs.09-4854 PMC3066624

[185] Rodriguez IR, Fliesler SJ (2009). Photodamage generates 7-keto- and 7-hydroxycholesterol in the rat retina via a free radical-mediated mechanism. Photochem Photobiol, 85:1116-1125. 19500292 10.1111/j.1751-1097.2009.00568.x PMC2793098

[186] Li Y, Song D, Song Y, Zhao L, Wolkow N, Tobias JW, et al. (2015). Iron-induced local complement component 3 (C3) up-regulation via non-canonical transforming growth factor (TGF)-beta signaling in the retinal pigment epithelium. J Biol Chem, 290:11918-11934. 25802332 10.1074/jbc.M115.645903 PMC4424331

[187] Gelfand BD, Wright CB, Kim Y, Yasuma T, Yasuma R, Li S, et al. (2015). Iron toxicity in the retina requires Alu RNA and the NLRP3 inflammasome. Cell Rep, 11:1686-1693. 26074074 10.1016/j.celrep.2015.05.023 PMC4481133

[188] Ananth S, Gnana-Prakasam JP, Bhutia YD, Veeranan-Karmegam R, Martin PM, Smith SB, et al. (2014). Regulation of the cholesterol efflux transporters ABCA1 and ABCG1 in retina in hemochromatosis and by the endogenous siderophore 2,5-dihydroxybenzoic acid. Biochim Biophys Acta, 1842:603-612. 24462739 10.1016/j.bbadis.2014.01.010 PMC4289134

[189] Anderson OA, Finkelstein A, Shima DT (2013). A2E induces IL-1ss production in retinal pigment epithelial cells via the NLRP3 inflammasome. PLoS One, 8:e67263. 23840644 10.1371/journal.pone.0067263 PMC3696103

[190] Kauppinen A, Niskanen H, Suuronen T, Kinnunen K, Salminen A, Kaarniranta K (2012). Oxidative stress activates NLRP3 inflammasomes in ARPE-19 cells-implications for age-related macular degeneration (AMD). Immunol Lett, 147:29-33. 22698681 10.1016/j.imlet.2012.05.005

[191] Liu RT, Gao J, Cao S, Sandhu N, Cui JZ, Chou CL, et al. (2013). Inflammatory mediators induced by amyloid-beta in the retina and RPE in vivo: implications for inflammasome activation in age-related macular degeneration. Invest Ophthalmol Vis Sci, 54:2225-2237. 23462752 10.1167/iovs.12-10849 PMC3947398

[192] Marneros AG (2013). NLRP3 inflammasome blockade inhibits VEGF-A-induced age-related macular degeneration. Cell Rep, 4:945-958. 24012762 10.1016/j.celrep.2013.08.002 PMC3821550

[193] Tarallo V, Hirano Y, Gelfand BD, Dridi S, Kerur N, Kim Y, et al. (2012). DICER1 loss and Alu RNA induce age-related macular degeneration via the NLRP3 inflammasome and MyD88. Cell, 149:847-859. 22541070 10.1016/j.cell.2012.03.036 PMC3351582

[194] Tseng WA, Thein T, Kinnunen K, Lashkari K, Gregory MS, D'Amore PA, et al. (2013). NLRP3 inflammasome activation in retinal pigment epithelial cells by lysosomal destabilization: implications for age-related macular degeneration. Invest Ophthalmol Vis Sci, 54:110-120. 23221073 10.1167/iovs.12-10655 PMC3544415

[195] Dodson M, Castro-Portuguez R, Zhang DD (2019). NRF2 plays a critical role in mitigating lipid peroxidation and ferroptosis. Redox Biol: 101107. 30692038 10.1016/j.redox.2019.101107 PMC6859567

[196] Yang WS, Stockwell BR (2008). Synthetic lethal screening identifies compounds activating iron-dependent, nonapoptotic cell death in oncogenic-RAS-harboring cancer cells. Chem Biol, 15:234-245. 18355723 10.1016/j.chembiol.2008.02.010 PMC2683762

[197] Yang WS, SriRamaratnam R, Welsch ME, Shimada K, Skouta R, Viswanathan VS, et al. (2014). Regulation of ferroptotic cancer cell death by GPX4. Cell, 156:317-331. 24439385 10.1016/j.cell.2013.12.010 PMC4076414

[198] Torii S, Shintoku R, Kubota C, Yaegashi M, Torii R, Sasaki M, et al. (2016). An essential role for functional lysosomes in ferroptosis of cancer cells. Biochem J, 473:769-777. 26759376 10.1042/BJ20150658

[199] Yang WS, Kim KJ, Gaschler MM, Patel M, Shchepinov MS, Stockwell BR (2016). Peroxidation of polyunsaturated fatty acids by lipoxygenases drives ferroptosis. Proc Natl Acad Sci U S A, 113:E4966-4975. 27506793 10.1073/pnas.1603244113 PMC5003261

[200] Gao M, Monian P, Quadri N, Ramasamy R, Jiang X (2015). Glutaminolysis and transferrin regulate ferroptosis. Mol Cell, 59:298-308. 26166707 10.1016/j.molcel.2015.06.011 PMC4506736

[201] Wang H, An P, Xie E, Wu Q, Fang X, Gao H, et al. (2017). Characterization of ferroptosis in murine models of hemochromatosis. Hepatology, 66:449-465. 28195347 10.1002/hep.29117 PMC5573904

[202] Kakhlon O, Cabantchik ZI (2002). The labile iron pool: characterization, measurement, and participation in cellular processes(1). Free Radic Biol Med, 33:1037-1046. 12374615 10.1016/s0891-5849(02)01006-7

[203] Cheng Z, Li Y (2007). What is responsible for the initiating chemistry of iron-mediated lipid peroxidation: an update. Chem Rev, 107:748-766. 17326688 10.1021/cr040077w

[204] Shah R, Shchepinov MS, Pratt DA (2018). Resolving the role of lipoxygenases in the initiation and execution of ferroptosis. ACS Cent Sci, 4:387-396. 29632885 10.1021/acscentsci.7b00589 PMC5879472

[205] Gao M, Monian P, Pan Q, Zhang W, Xiang J, Jiang X (2016). Ferroptosis is an autophagic cell death process. Cell Res, 26:1021-1032. 27514700 10.1038/cr.2016.95 PMC5034113

[206] Hou W, Xie Y, Song X, Sun X, Lotze MT, Zeh HJ 3rd, et al. (2016). Autophagy promotes ferroptosis by degradation of ferritin. Autophagy, 12:1425-1428. 27245739 10.1080/15548627.2016.1187366 PMC4968231

[207] Kwon MY, Park E, Lee SJ, Chung SW (2015). Heme oxygenase-1 accelerates erastin-induced ferroptotic cell death. Oncotarget, 6:24393-24403. 26405158 10.18632/oncotarget.5162 PMC4695193

[208] Masaldan S, Clatworthy SAS, Gamell C, Meggyesy PM, Rigopoulos AT, Haupt S, et al. (2018). Iron accumulation in senescent cells is coupled with impaired ferritinophagy and inhibition of ferroptosis. Redox Biol, 14:100-115. 28888202 10.1016/j.redox.2017.08.015 PMC5596264

[209] Reed JC, Pellecchia M (2012). Ironing out cell death mechanisms. Cell, 149:963-965. 22632964 10.1016/j.cell.2012.05.009

[210] Mancias JD, Wang X, Gygi SP, Harper JW, Kimmelman AC (2014). Quantitative proteomics identifies NCOA4 as the cargo receptor mediating ferritinophagy. Nature, 509:105-109. 24695223 10.1038/nature13148 PMC4180099

[211] Park E, Chung SW (2019). ROS-mediated autophagy increases intracellular iron levels and ferroptosis by ferritin and transferrin receptor regulation. Cell Death Dis, 10:822. 31659150 10.1038/s41419-019-2064-5 PMC6817894

[212] Lee JJ, Ishihara K, Notomi S, Efstathiou NE, Ueta T, Maidana D, et al. (2020). Lysosome-associated membrane protein-2 deficiency increases the risk of reactive oxygen species-induced ferroptosis in retinal pigment epithelial cells. Biochem Biophys Res Commun, 521:414-419. 31672277 10.1016/j.bbrc.2019.10.138 PMC6935401

[213] Cao JY, Dixon SJ (2016). Mechanisms of ferroptosis. Cell Mol Life Sci, 73:2195-2209. 27048822 10.1007/s00018-016-2194-1 PMC4887533

[214] Turchi R, Tortolici F, Guidobaldi G, Iacovelli F, Falconi M, Rufini S, et al. (2020). Frataxin deficiency induces lipid accumulation and affects thermogenesis in brown adipose tissue. Cell Death Dis, 11:51. 31974344 10.1038/s41419-020-2253-2 PMC6978516

[215] Doll S, Proneth B, Tyurina YY, Panzilius E, Kobayashi S, Ingold I, et al. (2017). ACSL4 dictates ferroptosis sensitivity by shaping cellular lipid composition. Nat Chem Biol, 13:91-98. 27842070 10.1038/nchembio.2239 PMC5610546

[216] Gao M, Jiang X (2018). To eat or not to eat-the metabolic flavor of ferroptosis. Curr Opin Cell Biol, 51:58-64. 29175614 10.1016/j.ceb.2017.11.001 PMC5949249

[217] Feng H, Stockwell BR (2018). Unsolved mysteries: how does lipid peroxidation cause ferroptosis? PLoS Biol, 16:e2006203. 29795546 10.1371/journal.pbio.2006203 PMC5991413

[218] Gaschler MM, Andia AA, Liu H, Csuka JM, Hurlocker B, Vaiana CA, et al. (2018). FINO2 initiates ferroptosis through GPX4 inactivation and iron oxidation. Nat Chem Biol, 14:507-515. 29610484 10.1038/s41589-018-0031-6 PMC5899674

[219] Seibt TM, Proneth B, Conrad M (2019). Role of GPX4 in ferroptosis and its pharmacological implication. Free Radic Biol Med, 133:144-152. 30219704 10.1016/j.freeradbiomed.2018.09.014

[220] Conrad M, Proneth B (2020). Selenium: Tracing another essential element of ferroptotic cell death. Cell Chem Biol, 27:409-419. 32275866 10.1016/j.chembiol.2020.03.012

[221] Bersuker K, Hendricks JM, Li Z, Magtanong L, Ford B, Tang PH, et al. (2019). The CoQ oxidoreductase FSP1 acts parallel to GPX4 to inhibit ferroptosis. Nature, 575:688-692. 31634900 10.1038/s41586-019-1705-2 PMC6883167

[222] Doll S, Freitas FP, Shah R, Aldrovandi M, da Silva MC, Ingold I, et al. (2019). FSP1 is a glutathione-independent ferroptosis suppressor. Nature, 575:693-698. 31634899 10.1038/s41586-019-1707-0

[223] Santoro MM (2020). The antioxidant role of non-mitochondrial CoQ10: mystery solved! Cell Metab, 31:13-15. 31951565 10.1016/j.cmet.2019.12.007

[224] Totsuka K, Ueta T, Uchida T, Roggia MF, Nakagawa S, Vavvas DG, et al. (2019). Oxidative stress induces ferroptotic cell death in retinal pigment epithelial cells. Exp Eye Res, 181:316-324. 30171859 10.1016/j.exer.2018.08.019 PMC7418497

[225] Conrad M, Angeli JP, Vandenabeele P, Stockwell BR (2016). Regulated necrosis: disease relevance and therapeutic opportunities. Nat Rev Drug Discov, 15:348-366. 26775689 10.1038/nrd.2015.6 PMC6531857

[226] Yoo SE, Chen L, Na R, Liu Y, Rios C, Van Remmen H, et al. (2012). Gpx4 ablation in adult mice results in a lethal phenotype accompanied by neuronal loss in brain. Free Radic Biol Med, 52:1820-1827. 22401858 10.1016/j.freeradbiomed.2012.02.043 PMC3341497

[227] Skouta R, Dixon SJ, Wang J, Dunn DE, Orman M, Shimada K, et al. (2014). Ferrostatins inhibit oxidative lipid damage and cell death in diverse disease models. J Am Chem Soc, 136:4551-4556. 24592866 10.1021/ja411006a PMC3985476

[228] Gascon S, Murenu E, Masserdotti G, Ortega F, Russo GL, Petrik D, et al. (2016). Identification and successful negotiation of a metabolic checkpoint in direct neuronal reprogramming. Cell Stem Cell, 18:396-409. 26748418 10.1016/j.stem.2015.12.003

[229] Chen L, Hambright WS, Na R, Ran Q (2015). Ablation of the ferroptosis inhibitor glutathione peroxidase 4 in neurons results in rapid motor neuron degeneration and paralysis. J Biol Chem, 290:28097-28106. 26400084 10.1074/jbc.M115.680090 PMC4653669

[230] Do Van B, Gouel F, Jonneaux A, Timmerman K, Gele P, Petrault M, et al. (2016). Ferroptosis, a newly characterized form of cell death in Parkinson's disease that is regulated by PKC. Neurobiol Dis, 94:169-178. 27189756 10.1016/j.nbd.2016.05.011

[231] Hambright WS, Fonseca RS, Chen L, Na R, Ran Q (2017). Ablation of ferroptosis regulator glutathione peroxidase 4 in forebrain neurons promotes cognitive impairment and neurodegeneration. Redox Biol, 12:8-17. 28212525 10.1016/j.redox.2017.01.021 PMC5312549

[232] Sakai O, Uchida T, Roggia MF, Imai H, Ueta T, Amano S (2015). Role of glutathione peroxidase 4 in glutamate-induced oxytosis in the retina. PLoS One, 10:e0130467. 26083388 10.1371/journal.pone.0130467 PMC4470664

[233] Roggia MF, Imai H, Shiraya T, Noda Y, Ueta T (2014). Protective role of glutathione peroxidase 4 in laser-induced choroidal neovascularization in mice. PLoS One, 9:e98864. 24897344 10.1371/journal.pone.0098864 PMC4045803

[234] Chew EY, Clemons TE, Agron E, Sperduto RD, Sangiovanni JP, Kurinij N, et al. (2013). Long-term effects of vitamins C and E, beta-carotene, and zinc on age-related macular degeneration: AREDS report no. 35. Ophthalmology, 120:1604-1611 e1604. 23582353 10.1016/j.ophtha.2013.01.021 PMC3728272

[235] van Leeuwen EM, Emri E, Merle BMJ, Colijn JM, Kersten E, Cougnard-Gregoire A, et al. (2018). A new perspective on lipid research in age-related macular degeneration. Prog Retin Eye Res, 67:56-86. 29729972 10.1016/j.preteyeres.2018.04.006

[236] Tate DJ Jr, Newsome DA, Oliver PD (1993). Metallothionein shows an age-related decrease in human macular retinal pigment epithelium. Invest Ophthalmol Vis Sci, 34:2348-2351. 8505216

[237] Liles MR, Newsome DA, Oliver PD (1991). Antioxidant enzymes in the aging human retinal pigment epithelium. Arch Ophthalmol, 109:1285-1288. 1929958 10.1001/archopht.1991.01080090111033

[238] Colak E, Zoric L, Radosavljevic A, Ignjatovic S (2018). The association of serum iron-binding proteins and the antioxidant parameter levels in age-related macular degeneration. Curr Eye Res, 43:659-665. 29448841 10.1080/02713683.2018.1437452

[239] Baksheeva VE, Tiulina VV, Tikhomirova NK, Gancharova OS, Komarov SV, Philippov PP, et al. (2018). Suppression of light-induced oxidative stress in the retina by mitochondria-targeted antioxidant. Antioxidants(Basel), 8. 10.3390/antiox8010003 PMC6356525 30577635

[240] Notomi S, Ishihara K, Efstathiou NE, Lee JJ, Hisatomi T, Tachibana T, et al. (2019). Genetic LAMP2 deficiency accelerates the age-associated formation of basal laminar deposits in the retina. Proc Natl Acad Sci U S A, 116:23724-23734. 31699817 10.1073/pnas.1906643116 PMC6876195

[241] Hadziahmetovic M, Song Y, Wolkow N, Iacovelli J, Grieco S, Lee J, et al. (2011). The oral iron chelator deferiprone protects against iron overload-induced retinal degeneration. Invest Ophthalmol Vis Sci, 52:959-968. 21051716 10.1167/iovs.10-6207 PMC4183363

[242] Song D, Zhao L, Li Y, Hadziahmetovic M, Song Y, Connelly J, et al. (2014). The oral iron chelator deferiprone protects against systemic iron overload-induced retinal degeneration in hepcidin knockout mice. Invest Ophthalmol Vis Sci, 55:4525-4532. 24970260 10.1167/iovs.14-14568 PMC4106252

[243] Lukinova N, Iacovelli J, Dentchev T, Wolkow N, Hunter A, Amado D, et al. (2009). Iron chelation protects the retinal pigment epithelial cell line ARPE-19 against cell death triggered by diverse stimuli. Invest Ophthalmol Vis Sci, 50:1440-1447. 19182262 10.1167/iovs.08-2545 PMC2665187

[244] Ceci A, Felisi M, De Sanctis V, De Mattia D (2003). Pharmacotherapy of iron overload in thalassaemic patients. Expert Opin Pharmacother, 4:1763-1774. 14521486 10.1517/14656566.4.10.1763

[245] Shirai Y, Mori A, Nakahara T, Sakamoto K, Ishii K (2015). Deferiprone protects against photoreceptor degeneration induced by tunicamycin in the rat retina. Biol Pharm Bull, 38:1076-1080. 26133718 10.1248/bpb.b15-00185

[246] Song D, Dunaief JL (2013). Retinal iron homeostasis in health and disease. Front Aging Neurosci, 5:24. 23825457 10.3389/fnagi.2013.00024 PMC3695389

[247] Neufeld EJ (2006). Oral chelators deferasirox and deferiprone for transfusional iron overload in thalassemia major: new data, new questions. Blood, 107:3436-3441. 16627763 10.1182/blood-2006-02-002394 PMC1895765

[248] Shi L, Ito F, Wang Y, Okazaki Y, Tanaka H, Mizuno M, et al. (2017). Non-thermal plasma induces a stress response in mesothelioma cells resulting in increased endocytosis, lysosome biogenesis and autophagy. Free Radic Biol Med, 108:904-917. 28465262 10.1016/j.freeradbiomed.2017.04.368

[249] Kalinowski DS, Richardson DR (2005). The evolution of iron chelators for the treatment of iron overload disease and cancer. Pharmacol Rev, 57:547-583. 16382108 10.1124/pr.57.4.2

[250] Hidajat RR, McLay JL, Goode DH, Spearing RL (2004). EOG as a monitor of desferrioxamine retinal toxicity. Doc Ophthalmol, 109:273-278. 15957612 10.1007/s10633-005-1336-9

[251] Wu CH, Yang CP, Lai CC, Wu WC, Chen YH (2014). Deferoxamine retinopathy: spectral domain-optical coherence tomography findings. BMC Ophthalmol, 14:88. 24989140 10.1186/1471-2415-14-88 PMC4090392

[252] Lu M, Hansen RM, Cunningham MJ, Eklund SE, Fulton AB (2007). Effects of desferoxamine on retinal and visual function. Arch Ophthalmol, 125:1581-1582. 17998528 10.1001/archopht.125.11.1581

[253] Karimi M, Asadi-Pooya AA, Khademi B, Asadi-Pooya K, Yarmohammadi H (2002). Evaluation of the incidence of sensorineural hearing loss in beta-thalassemia major patients under regular chelation therapy with desferrioxamine. Acta Haematol, 108:79-83. 12187025 10.1159/000064748

[254] Di Nicola M, Barteselli G, Dell'Arti L, Ratiglia R, Viola F (2015). Functional and structural abnormalities in deferoxamine retinopathy: a review of the literature. Biomed Res Int, 2015:249617. 26167477 10.1155/2015/249617 PMC4475708

[255] Baath JS, Lam WC, Kirby M, Chun A (2008). Deferoxamine-related ocular toxicity: incidence and outcome in a pediatric population. Retina, 28:894-899. 18536609 10.1097/IAE.0b013e3181679f67

[256] Charkoudian LK, Pham DM, Franz KJ (2006). A pro-chelator triggered by hydrogen peroxide inhibits iron-promoted hydroxyl radical formation. J Am Chem Soc, 128:12424-12425. 16984186 10.1021/ja064806w

[257] Chevion M (1991). Protection against free radical-induced and transition metal-mediated damage: the use of "pull" and "push" mechanisms. Free Radic Res Commun, 12-13 Pt 2:691-696. 10.3109/10715769109145848 2060841

[258] Obolensky A, Berenshtein E, Lederman M, Bulvik B, Alper-Pinus R, Yaul R, et al. (2011). Zinc-desferrioxamine attenuates retinal degeneration in the rd10 mouse model of retinitis pigmentosa. Free Radic Biol Med, 51:1482-1491. 21824515 10.1016/j.freeradbiomed.2011.07.014

[259] Sakamoto K, Suzuki T, Takahashi K, Koguchi T, Hirayama T, Mori A, et al. (2018). Iron-chelating agents attenuate NMDA-Induced neuronal injury via reduction of oxidative stress in the rat retina. Exp Eye Res, 171:30-36. 29530811 10.1016/j.exer.2018.03.008

[260] Kontoghiorghes GJ, Neocleous K, Kolnagou A (2003). Benefits and risks of deferiprone in iron overload in Thalassaemia and other conditions: comparison of epidemiological and therapeutic aspects with deferoxamine. Drug Saf, 26:553-584. 12825969 10.2165/00002018-200326080-00003

[261] Sohn YS, Breuer W, Munnich A, Cabantchik ZI (2008). Redistribution of accumulated cell iron: a modality of chelation with therapeutic implications. Blood, 111:1690-1699. 17975016 10.1182/blood-2007-07-102335

[262] Cappellini MD, Pattoneri P (2009). Oral iron chelators. Annu Rev Med, 60:25-38. 19630568 10.1146/annurev.med.60.041807.123243

[263] Glickstein H, El RB, Shvartsman M, Cabantchik ZI (2005). Intracellular labile iron pools as direct targets of iron chelators: a fluorescence study of chelator action in living cells. Blood, 106:3242-3250. 16020512 10.1182/blood-2005-02-0460

[264] Hadziahmetovic M, Pajic M, Grieco S, Song Y, Song D, Li Y, et al. (2012). The oral iron chelator deferiprone protects against retinal degeneration induced through diverse mechanisms. Transl Vis Sci Technol, 1:7. 24049707 10.1167/tvst.1.2.7 PMC3763881

[265] Kontoghiorghes GJ (2007). Deferasirox: uncertain future following renal failure fatalities, agranulocytosis and other toxicities. Expert Opin Drug Saf, 6:235-239. 17480173 10.1517/14740338.6.3.235

[266] Fisher SA, Brunskill SJ, Doree C, Chowdhury O, Gooding S, Roberts DJ (2013). Oral deferiprone for iron chelation in people with thalassaemia. Cochrane Database Syst Rev:CD004839. 23966105 10.1002/14651858.CD004839.pub3 PMC11843083

[267] Kontoghiorghes GJ, Pattichi K, Hadjigavriel M, Kolnagou A (2000). Transfusional iron overload and chelation therapy with deferoxamine and deferiprone (L1). Transfus Sci, 23:211-223. 11099897 10.1016/s0955-3886(00)00089-8

[268] Brittenham GM (2011). Iron-chelating therapy for transfusional iron overload. N Engl J Med, 364:146-156. 21226580 10.1056/NEJMct1004810 PMC3078566

[269] Pan Y, Keane PA, Sadun AA, Fawzi AA (2010). Optical coherence tomography findings in deferasirox-related maculopathy. Retin Cases Brief Rep, 4:229-232. 25390664 10.1097/ICB.0b013e3181af7b44

[270] Song D, Song Y, Hadziahmetovic M, Zhong Y, Dunaief JL (2012). Systemic administration of the iron chelator deferiprone protects against light-induced photoreceptor degeneration in the mouse retina. Free Radic Biol Med, 53:64-71. 22579919 10.1016/j.freeradbiomed.2012.04.020 PMC3380452

[271] Horackova M, Ponka P, Byczko Z (2000). The antioxidant effects of a novel iron chelator salicylaldehyde isonicotinoyl hydrazone in the prevention of H(2)O(2) injury in adult cardiomyocytes. Cardiovasc Res, 47:529-536. 10963725 10.1016/s0008-6363(00)00088-2

[272] Simunek T, Boer C, Bouwman RA, Vlasblom R, Versteilen AM, Sterba M, et al. (2005). SIH--a novel lipophilic iron chelator--protects H9c2 cardiomyoblasts from oxidative stress-induced mitochondrial injury and cell death. J Mol Cell Cardiol, 39:345-354. 15978614 10.1016/j.yjmcc.2005.05.008

[273] Klimtova I, Simunek T, Mazurova Y, Kaplanova J, Sterba M, Hrdina R, et al. (2003). A study of potential toxic effects after repeated 10-week administration of a new iron chelator--salicylaldehyde isonicotinoyl hydrazone (SIH) to rabbits. Acta Medica (Hradec Kralove), 46:163-170. 14965167

[274] Kurz T, Karlsson M, Brunk UT, Nilsson SE, Frennesson C (2009). ARPE-19 retinal pigment epithelial cells are highly resistant to oxidative stress and exercise strict control over their lysosomal redox-active iron. Autophagy, 5:494-501. 19223767 10.4161/auto.5.4.7961

[275] Charkoudian LK, Dentchev T, Lukinova N, Wolkow N, Dunaief JL, Franz KJ (2008). Iron prochelator BSIH protects retinal pigment epithelial cells against cell death induced by hydrogen peroxide. J Inorg Biochem, 102:2130-2135. 18835041 10.1016/j.jinorgbio.2008.08.001 PMC2662444

[276] Caro AA, Commissariat A, Dunn C, Kim H, Garcia SL, Smith A, et al. (2015). Prooxidant and antioxidant properties of salicylaldehyde isonicotinoyl hydrazone iron chelators in HepG2 cells. Biochim Biophys Acta, 1850:2256-2264. 26275495 10.1016/j.bbagen.2015.08.005 PMC4587295

[277] Zhao L, Wang C, Song D, Li Y, Song Y, Su G, et al. (2014). Systemic administration of the antioxidant/iron chelator alpha-lipoic acid protects against light-induced photoreceptor degeneration in the mouse retina. Invest Ophthalmol Vis Sci, 55:5979-5988. 25146987 10.1167/iovs.14-15025 PMC4172298

[278] Picard E, Jonet L, Sergeant C, Vesvres MH, Behar-Cohen F, Courtois Y, et al. (2010). Overexpressed or intraperitoneally injected human transferrin prevents photoreceptor degeneration in rd10 mice. Mol Vis, 16:2612-2625. 21179240 PMC3002967

[279] Picard E, Le Rouzic Q, Oudar A, Berdugo M, El Sanharawi M, Andrieu-Soler C, et al. (2015). Targeting iron-mediated retinal degeneration by local delivery of transferrin. Free Radic Biol Med, 89:1105-1121. 26454080 10.1016/j.freeradbiomed.2015.08.018

[280] Tanito M, Nishiyama A, Tanaka T, Masutani H, Nakamura H, Yodoi J, et al. (2002). Change of redox status and modulation by thiol replenishment in retinal photooxidative damage. Invest Ophthalmol Vis Sci, 43:2392-2400. 12091442

[281] Wood JP, Pergande G, Osborne NN (1998). Prevention of glutathione depletion-induced apoptosis in cultured human RPE cells by flupirtine. Restor Neurol Neurosci, 12:119-125. 12671306

[282] Jin M, Yaung J, Kannan R, He S, Ryan SJ, Hinton DR (2005). Hepatocyte growth factor protects RPE cells from apoptosis induced by glutathione depletion. Invest Ophthalmol Vis Sci, 46:4311-4319. 16249513 10.1167/iovs.05-0353

[283] Armstrong JS, Whiteman M, Yang H, Jones DP, Sternberg P Jr, (2004). Cysteine starvation activates the redox-dependent mitochondrial permeability transition in retinal pigment epithelial cells. Invest Ophthalmol Vis Sci, 45:4183-4189. 15505073 10.1167/iovs.04-0570

[284] Lewerenz J, Ates G, Methner A, Conrad M, Maher P (2018). Oxytosis/ferroptosis-(re-) emerging roles for oxidative stress-dependent non-apoptotic cell death in diseases of the central nervous system. Front Neurosci, 12:214. 29731704 10.3389/fnins.2018.00214 PMC5920049

[285] Marchette LD, Thompson DA, Kravtsova M, Ngansop TN, Mandal MN, Kasus-Jacobi A (2010). Retinol dehydrogenase 12 detoxifies 4-hydroxynonenal in photoreceptor cells. Free Radic Biol Med, 48:16-25. 19686838 10.1016/j.freeradbiomed.2009.08.005 PMC2874966

[286] Kim J, Cho K, Choung SY (2019). Protective effect of Prunella vulgaris var. L extract against blue light induced damages in ARPE-19 cells and mouse retina. Free Radic Biol Med, 152:622-631. 31811921 10.1016/j.freeradbiomed.2019.12.003

